# Transoral Robotic Cleft Palate Surgery: Communication-Related Outcomes and Feasibility

**DOI:** 10.3390/s26134308

**Published:** 2026-07-07

**Authors:** Tim Frederik Peter Ritzen, Jill Goris, Lisa E. Ramaut, Darren I. Booi, René R. W. J. van der Hulst, Moustapha Hamdi, Nasser Nadjmi, Rutger M. Schols

**Affiliations:** 1 Department of Plastic, Reconstructive and Hand Surgery, Cleft Team, Maastricht University Medical Centre, MosaKids Children’s Hospital, P. Debyelaan 25, 6229 HX Maastricht, The Netherlands; 2 Faculty of Health, Medicine and Life Sciences, Maastricht University, Universiteitssingel 40, 6229 ER Maastricht, The Netherlands; 3 Department of Plastic, Reconstructive and Aesthetic Surgery, Cleft Team, University Hospital Brussels, KidzHealthCastle Children’s Hospital, Brussels Health Campus, Laarbeeklaan 101, 1090 Brussels, Belgium; 4 Faculty of Medicine & Pharmacy, Free University Brussels, Brussels Health Campus, Laarbeeklaan 103, 1090 Brussels, Belgium; 5 Department of Cranio-Maxillofacial Surgery, Cleft Team, Antwerp University Hospital, Drie Eikenstraat 665, 2650 Antwerp, Belgium; 6 Faculty of Medicine & Health Sciences, University of Antwerp, Prinsstraat 13, 2000 Antwerp, Belgium

**Keywords:** transoral robotic cleft surgery (TORCS), robot-assisted surgery, cleft palate repair, communication, structured literature review

## Abstract

Treatment of cleft lip alveolus and/or palate includes surgical repair to treat communication-related outcomes such as velopharyngeal insufficiency and otological dysfunction. Robot-assisted surgery has recently evolved into a promising adjunct to conventional surgery, particularly for complex procedures such as transoral (reconstructive) surgery. This structured literature review aims to investigate whether robot-assisted transoral cleft palate repair enhances communication (i.e., speech and otological) outcomes compared to conventional manual cleft palate surgery. A literature search was performed using PubMed, Embase, the Cochrane Library and Google Scholar. Primary outcomes were the change in cleft speech characteristics and otological disease after robot-assisted cleft palate surgery versus manual cleft palate surgery. The available evidence on communication-related outcomes remains sparse. Six relevant articles were included. In only one study, transoral robotic cleft surgery (TORCS) significantly reduced otitis media with effusion (OME), need for ventilation tubes and hearing threshold within 2 years post-surgery. No postoperative speech or velopharyngeal outcomes were reported. In conclusion, transoral robotic cleft surgery (TORCS) appears safe and feasible for repair of a cleft palate. It provides superior intraoral view and improved surgeon ergonomics. Current drawbacks are the costs and the available tools, the extended surgical duration and the lack of haptic feedback, which limit the current clinical applicability of TORCS. Based on limited clinical evidence, TORCS may support faster recovery of Eustachian tube function and hearing, but no conclusions on speech outcomes can yet be drawn.

## 1. Introduction

Cleft lip alveolus and/or palate represents one of the most prevalent congenital anomalies in Belgium and the Netherlands, with an estimated annual prevalence of 14.7 and 16.6 cases per 10,000 live births respectively [1,2].

The main goal of surgical cleft palate repair is closure of the oral and nasal cavities, thereby restoring normal anatomy for competent velopharyngeal function [3,4,5,6]. Robot-assisted surgery has recently evolved into a promising adjunct to conventional surgery, particularly in the context of complex and technically demanding procedures, such as transoral (reconstructive) surgery. In selected surgical fields, robotic systems have been associated with improved visualisation, precision, and ergonomics, although their benefits remain procedure-specific [7,8,9,10]. Hockstein et al. already described that robot-assisted surgery is possible in the laryngeal and pharyngeal area [11,12]. Collectively, these findings open important avenues for future research, particularly whether robot-assisted transoral cleft palate repair enhances communication-related outcomes such as velopharyngeal insufficiency and otological dysfunction.

Velopharyngeal insufficiency leads to hypernasal speech, nasal emission and weak pressure consonants [13,14,15]. It requires intensive speech therapy combined with surgical treatment in 20–30% of the cases [4]. Being present in 97% of all cleft patients, otitis media with or without effusion is the most frequent otological problem in this population and could persist even after palatoplasty [16,17]. Chronic middle ear infections with effusion lead to conductive hearing loss in cleft patients, negatively affecting their communication [18,19,20].

Given the technical challenges inherent to cleft surgery and the scientific and technological progress in the field of robotic surgery, there is a clear need for research into its potential applications in this domain. This structured literature review therefore aims to investigate whether transoral robot-assisted cleft palate surgery enhances communication (i.e., speech and otological) outcomes compared to conventional manual cleft palate repair.

## 2. Materials and Methods

Ethical approval was not required because this study was based exclusively on published literature.

### 2.1. Literature Search

An elaborate electronic database literature search was performed independently by two authors (TR and JG) on 19 January 2026 and 25 January 2026. Although no formal review protocol was registered, the objectives, inclusion and exclusion criteria, search methodology, and study selection procedures were defined prior to conducting the literature search and screening. PubMed, Embase and the Cochrane Library were used. Google Scholar was used as an additional method for literature search. The only search limit used in both the database and Google Scholar search was that articles were written in Dutch or English. Because clinical TORCS literature is sparse, preclinical studies were included to map feasibility, instrumentation, and technical barriers, while communication outcomes were expected only from clinical studies. In addition, studies comparing robotic and conventional (manual) cleft surgery were included. We primarily focused on outcomes related to communication, including hearing impairment and velopharyngeal insufficiency-associated speech difficulties. Both animal and human studies were considered. Systematic or literature reviews were excluded. Key search terms, which included “Robotic cleft palate surgery”, “Cleft palate”, “Velopharyngeal insufficiency associated speech difficulties”, “Audiological disease” and “Manual cleft palate surgery” were combined by use of various combinations of Boolean operators “OR” and “AND”.

Initially, the search was conducted using a combination of all search terms. This approach yielded a very limited number of articles; therefore, it was necessary to broaden the search strategy. Consequently, alternative combinations of search terms were used.

The reference lists of primarily included articles were additionally screened for relevant studies. The database search strategies can be found in Supplemental Tables S1–S3.

### 2.2. Study Selection

Using EndNote 2025 (Version 2025.3.1; Clarivate Plc, Philadelphia, PA, USA), the retrieved articles were independently assessed for duplicates. All articles were screened on title and abstract, based on the in- and exclusion criteria. For the remaining articles, the full texts were obtained and independently reviewed by two authors (TR and JG). Disagreements were settled by consensus or a third reviewer (RS). The PRISMA flow chart on the selection process can be found in Supplemental Figure S1. An overview of the included and excluded publications is provided in Supplemental Material S1.

### 2.3. Data Extraction

Data from each selected article was extracted by TR and JG and collected in Microsoft Excel for Microsoft 365 (Version 2603, Build 19822.20142; Microsoft Corp., Redmond, WA, USA). 

### 2.4. Primary Outcome Measures

Primary outcomes were the change in cleft speech characteristics and otological disease after surgical cleft repair compared between robot-assisted and manual cleft palate surgery. The extracted data included results of (perceptual) speech assessments, (the presence, type and degree of) hearing loss, occurrence of otitis media, and ventilation tube placement.

### 2.5. Secondary Outcome Measures

Secondary outcomes included the presence of surgical complications, such as bleeding, wound dehiscence, fistula or bifid uvula. In addition, data on the safety and feasibility of robot-assisted cleft palate surgery, cleft surgeon ergonomics, surgery duration and preference for surgical technique were gathered.

### 2.6. Methodological Quality Assessment

A methodological quality assessment was performed using the IDEAL framework and the Robins-I tool for risk of bias assessment [21,22,23]. The complete methodological quality assessment can be found in Supplemental Material S2.

## 3. Results

### 3.1. Baseline Characteristics

This study included six articles published between 2013 and 2025 of which four were preclinical [24,25,26,27], one was fully clinical [28] and one was mixed preclinical/clinical [29]. No included study reported on postoperative speech outcomes following TORCS in primary cleft palate repair. Only one included study addressed otological outcomes after TORCS. In the preclinical setting, specially crafted cleft models or fresh human cadaveric heads were used for the transoral robotic (cleft) surgery (TOR(C)S) simulations. In total, nine simulations were carried out on either a model or a cadaver.

Both clinical studies included cleft patients [28,29]. TORCS was performed in 39 cleft patients who were matched with 53 control cleft patients.

The surgical techniques used included a posterior pharyngeal flap (PPF), modified Furlow palatoplasty, Hynes Pharyngoplasty and two-flap palatoplasty (e.g., Von Langenbeck palatoplasty). An overview of the included studies’ characteristics is provided in Table 1.

### 3.2. Methodological Quality Assessment

**Figure 1:**
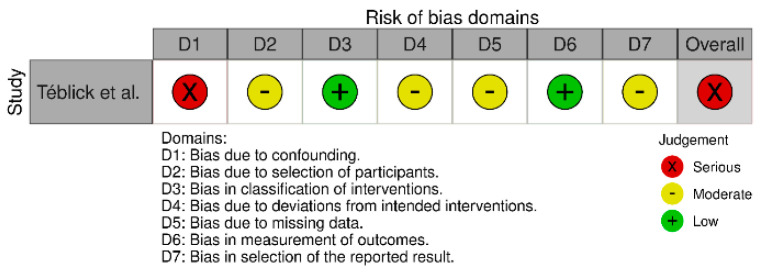
Risk of bias assessment for the study by Téblick et al. [28] using the ROBINS-I tool. D1–D7 represent the specific bias domains; symbols indicate the judgement for each domain and the overall risk (green/+: low; yellow/−: moderate; red/X: serious).

The included preclinical studies successfully satisfy the mandates of IDEAL Stages 0, 1 and 2a by proving that robotic systems can safely navigate and reconstruct the velopharyngeal mechanism [21]. However, the current body of evidence is methodologically insufficient to claim clinical superiority over conventional manual palatoplasty. An overview of the most important endpoints of the methodological quality assessment is presented in Table 2. As shown in Figure 1, the clinical study by Téblick et al. [28] has a serious risk of bias, according to the ROBINS-1 tool [22,23].

### 3.3. Clinical Communication Outcomes

#### Postoperative Communication Outcomes

The study by Téblick et al. [28] is the only study that investigated communication after TORCS. They particularly looked at middle ear function after usage of the Da Vinci robot (Intuitive Surgical Inc., Sunnyvale, CA, USA) during soft palate closure with a modified Furlow double-opposing Z-palatoplasty. Therefore, the following outcome parameters were used: otitis media with effusion (OME), tympanostomy tube use, and hearing loss during 2 years of follow-up. At 2 years post-surgery, the percentage of children with OME had reduced significantly to 30% in the manual group and to 10% in the robot group. The need for ventilation tubes (VTs) decreased significantly over time, with fewer children needing new VTs during postoperative follow-up in the robot group (41%) than in the manual group (91%) (*p* = 0.026). Regarding hearing loss, significantly lower hearing thresholds were recorded in the robot group from 7 to 18 months postoperatively (7–12 months; *p*  =  0.017) and (13–18 months; *p*  =  0.041).

### 3.4. Preclinical Feasibility and Technical Findings

#### 3.4.1. Collision Analyses

Three studies paid attention to the collisions of instruments with other instruments and the oral cavity.

Podolsky et al. [26] observed and compared tool–cavity collisions between the Da Vinci Si and Xi systems for the majority of the surgical steps. The Da Vinci Si system had an inferior performance compared to the Xi system as it showed more tool–cavity collisions overall (in 11 versus nine steps) and severe collisions causing significant deformation of the cleft model (in four versus zero steps, respectively).

Maguire et al. [27] looked closely into the aetiology of tool–tool and tool–cavity collisions. The fewest collisions occurred when operating the central part of the palate. Due to the geometry of the palate reachability during suturing, which decreases towards the outer anterior and especially towards the posterior limits of the palate, the frequency of collisions in those areas increases. This difference in reachability between the anterior and posterior palates was observed in both tool–tool and tool–cavity collisions but was only significant for tool–cavity collisions. Furthermore, positioning the trocar at a higher level relative to the patient’s body may increase instrument collisions, as longer tool shafts are required. The increased length from the remote centre of motion of the trocar to the palate surface results in exaggerated movements at the level of the oral cavity, increasing the risk of tool–cavity collisions. Finally, comparing 3 mm instruments to 8 mm instruments (as used in Khan et al. and Podolsky et al.), this study found that the use of 3 mm tools resulted in both reduced tool–cavity collisions (0.5 versus 7.67 average collisions per suture) and reduced tool–tool collisions (0.67 versus 2.00 average collisions per suture) per suture, compared to the 8 mm tools.

Khan et al. [25] also recognise these collisions but state “they were not of sufficient force to cause any soft-tissue injury or to rotate the head.”

#### 3.4.2. Operative Times

Surgical duration was reported in four studies [24,26,28,29], but Téblick et al. and Nadjmi et al. [28,29] report on outcomes of surgery in the same centre and performed by the same surgeon. In those clinical studies, a mean TORCS duration of 122 ± 8 min was reported, but it was not specified which steps in the surgical process, e.g., anaesthesia, preparation of the robot, dissection, etc., were included. Podolsky et al. and Smartt et al. [24,26], the only included preclinical studies reporting on operating time, found a surgical duration of 180 to 240 min respectively and a mean of 113 min (105, 113 and 122 min). Nadjmi et al. [29] was the only study that compared surgical duration between TORCS and conventional manual cleft surgery. As the mean operating time for manual cleft repair was reported to be 87 ± 6 min, this manual approach was statistically significantly faster.

## 4. Discussion

This structured literature review aimed to assess the effectiveness of robot-assisted cleft palate surgery compared to manual cleft palate repair in the treatment of communication disorders (i.e., speech and otological problems) in cleft patients. Transoral robotic surgery (TORS) has been widely researched, whilst specifically transoral robotic cleft surgery (TORCS) has been less frequently studied.

Six relevant articles were included discussing TOR(C)S using surgical techniques that are common in cleft care. Unfortunately, comparative data is rather limited. Five publications mainly focus on the feasibility of TORS using different system set-ups, instruments, surgical techniques, or practice models [24,25,26,27,29]. The one remaining article focuses on restoration of Eustachian tube function after cleft repair and its effect on otological outcomes [28]. No other communication outcomes, such as speech or velopharyngeal insufficiency, have been researched throughout the other articles, making it impossible to draw meaningful conclusions regarding the effect of TORCS on overall communication outcomes compared with manual cleft palate repair.

### 4.1. TORCS and Communication

The only included study reporting on otological outcomes was Téblick et al. [28]. Here TORCS was able to provide a clearer image of the palatal structures, allowing for a more precise dissection. They argue that this accurate dissection leads to a better muscle reconstruction and has been one of the main causes for a faster recovery of the Eustachian tube function. Compared to manual cleft surgery, TORCS showed a decrease in OME (30% vs. 10% respectively at 2 years post-surgery), a decrease in the need for ventilation tubes (91% vs. 41% respectively (*p* = 0.026)) and significantly lower hearing thresholds (7–12 months; *p*  =  0.017 and 13–18 months; *p*  =  0.041). Since there are currently no other studies conducted on otological outcomes after TORCS, there is no existing literature to compare these results to. More research is therefore needed to investigate communication-related outcomes after robotic cleft repair.

### 4.2. Preferred Set-Up of Robotic System

**Figure 2:**
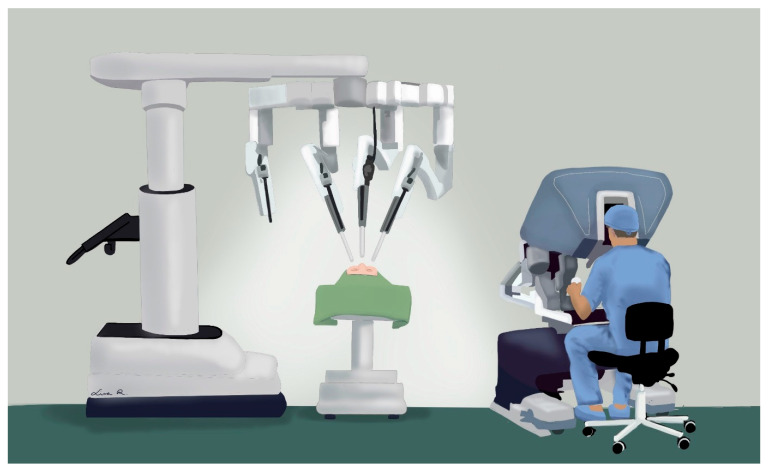
Overview of TORCS set-up. A schematic depiction of the operative set-up in transoral robotic cleft surgery (TORCS), illustrating the positioning of the robotic surgical platform adjacent to the operating table and its relation to the surgical field.

**Figure 3:**
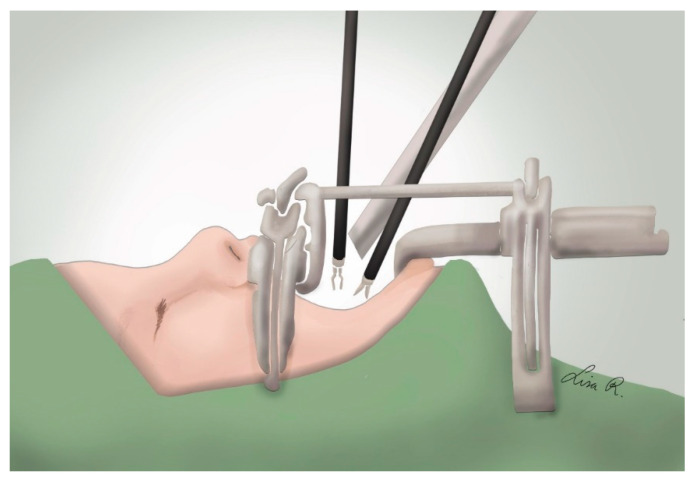
Close-up TORCS set-up. A schematic close-up illustration of TORCS, showing a Dingman retractor being placed to hold the maxilla and mandible apart while robotic surgical instruments are inserted into the oral cavity.

In the reviewed articles, we identified that the use of a 30-degree endoscopic camera together with two instruments is preferred. Whilst the patient is being intubated on one side of the operating table, the robot can be prepared on the other side by the surgeon and assistants. After intubation and turning the table 180 degrees, the patient is ideally placed in a 0-degree supine position with his head slightly extended like in manual surgery. A Dingman retractor can be successfully used to provide an optimal intraoral view [24,25,26,27,29]. As the surgeon is seated at a remote console, the surgical assistant can be conveniently seated near the head of the patient. See Figure 2 and Figure 3 for an illustration of the described robotic set-up in the operating theatre.

All studies have used the Da Vinci robotic surgery system, varying between the Da Vinci Si, Xi, S or an unspecified system. Two studies [26,27] compared different system mechanics, like those of the Da Vinci Si to Xi, in their feasibility for TORCS. As not all studies reported the type of Da Vinci system used, no definite conclusion can be drawn on the optimal system.

### 4.3. Advantages of TORCS Compared to Manual Surgical Cleft Palate Repair

The most frequently mentioned advantage of TORS is the superior 3D endoscopic view during surgery resulting in a more precise dissection and probably in superior outcomes. During manual cleft palate surgery, surgeons often use magnifying glasses or a conventional surgical microscope in order to ensure a clear view of the different anatomical structures. Mokhtar et al. [8] have recently published an article on robotic microscope (RoboticScope, BHS Technologies, Inc., Innsbruck, Austria) enhanced cleft palate surgery, using a head mounted display connected to a hands-free head-controlled robotic guided surgical microscope. On top of enhancing surgical precision, through improved visualisation and ergonomics like conventional surgical microscopes, this microscope further improves surgeon posture and surgical workflow due to its hands-free controls. This technology potentially offers a more pragmatic and clinically feasible interim solution relative to complete robotic transoral surgery. Selber et al. [30] even argues that the robotic endoscope has inferior optics compared to the robotic microscope. However, according to the authors of the included clinical studies [26,28,29], the surgical microscopes remain inferior to the robotic view and still result in worse ergonomics for the surgeon. This is further supported by an included preclinical study [26,28,29]. The use of the robotic system provides the surgeon with a clear view while seated at the console in an ergonomically favourable position, as has been demonstrated in multiple studies [12,24,25,26,29].

Furthermore, as the robot needs to translate the large movements of the surgeon’s hands to the smaller movements of the robotic instruments, subtle tremors are filtered out allowing for better-coordinated movements compared to manual surgery [28,29].

### 4.4. Disadvantages of TORCS Compared to Manual Surgical Cleft Repair

Surgical duration of manual cleft surgery (80 to 153 min) [31,32,33] is significantly shorter than in TORCS (105 and 240 min) [24,26,28,29]. It is however not always very clear if the reported robotic operating times include anaesthetising and preparation of the patient or solely the surgical cleft repair. Moreover, as observed in other surgical disciplines, the additional operative time associated with the adoption of robotic surgery is expected to decrease as the surgical team gains experience. The included studies reported that this experience could be gained especially in preparation of the patient within the robotic system. Smartt et al. demonstrated a steep learning curve in the use of the robotic system; however, a true decrease in operative time is not described [24].

Nevertheless, due to the increased duration of surgery together with the expensive, limited available equipment, space in the operating room and training needed, performing TORCS remains very expensive. Importantly, its clinical applicability in paediatric cleft surgery could be further limited by challenges in airway management [34]. The equipment that is currently available for TORS is being adapted beyond its original intended indications. Although it is suitable, the equipment is truly designed for abdominal endoscopic procedures. Therefore, it has features that limit efficient transoral surgery, particularly in paediatric patients with a restricted intraoral working space.

In some centres, the robotic microscope could therefore serve as a good interim solution as the conventional manual instruments can be used, surgical duration is minimally elongated by an average of 28 min compared to manual cleft surgery, and surgeon ergonomics are evidently improved [8]. Dedicated microsurgical robots such as MUSA (Microsure B.V., Eindhoven, The Netherlands) and Symani Surgical System (Medical Microinstruments, Inc., Wilmington, DE, USA), that are being used in combination with a conventional microscope or exoscope [29,30]. These new robotic systems may open new possibilities for surgeons and hospitals to put together robotic workflows, tailored to their budget, needs and preferences.

Finally, authors frequently report the lack of haptic feedback as an important safety issue. During manual cleft surgery, the amount of pressure put on the instruments by the surgeons deliberately or by structures in the oral cavity can be felt through the instruments. In the current TORCS, the robot does not forward these feelings to the surgeon who is therefore ignorant of potential risks such as ruptures within the oral cavity. This is reported in a clinical study, as well as in preclinical studies [24,26,29]. Especially in the paediatric cleft population, feedback through sensation is important for careful dissection and preservation of the vascular pedicle of the palatal flaps. Excessive stretching or compression of the delicate tissues could damage this pedicle and result in flap necrosis [35]. Haptic feedback is however being integrated into the development of new robotic systems. The Da Vinci 5 (Intuitive Surgical, Inc., Sunnyvale, CA, USA) system enables surgeons to sense the push and pull at the instrument tips through its Force Feedback technology. With haptic feedback, the exerted force is expected to be reduced by 20–43% which could ensure an even safer dissection of the intraoral structures [36,37]. However, mainly novice surgeons acknowledge the value of this Force Feedback technology whilst high-volume surgeons do not find it useful for application in their own practice [38].

### 4.5. Safety of TORCS

In a study by Hockstein et al. [11], the possible risk of tool–cavity collisions was researched by attempting to deliberately mutilate fresh human cadaveric heads. This research showed the robotic tools could only cause superficial injuries but no significant injuries, suggesting that clinically significant injury is unlikely. This was confirmed by the included clinical trials reporting no major complications due to these collisions [28,29]. Another clinical trial on TORS and oropharyngeal malignancies yielded comparable results, thereby reinforcing these findings even more [39]. Furthermore, bones or teeth could not be fractured, meaning TORS could also be used in secondary or revision surgery in older children who have already developed teeth [11].

Moreover, the set-up of TORS allows the surgical assistant to be at the head of the patient while the surgeon works at the console. This is convenient as they are needed for suctioning, but they can also simultaneously keep an eye on possible dangerous collisions [24,25,26,29].

Various studies included have researched the ideal diameter and articulating joints in the available equipment. The 5 and 8 mm instruments currently available have shown no true superiority, but the use of the 5 mm instruments could occasionally advantageously include a third instrument [26]. Articles have described tool–tool and tool–oral cavity collisions for both 5 mm and 8 mm instruments and one study has compared 3 mm to 8 mm instruments. The 3 mm tools had fewer tool–cavity collisions compared to the 8 mm instruments and, because of their smaller diameter, resulted in improved visualisation of the surgical field [27].

Hence, it seems beneficial to use instruments with the smallest diameter available to increase safety, visualisation and the number of tools that can be used in the oral cavity.

### 4.6. Future Perspective

Current literature suggests a safe application of TORCS in clinical practice but does not show clinical treatment outcomes. This important clinical evidence gap must therefore be addressed in future research. Although conventional cleft palate surgery achieves excellent functional outcomes in experienced cleft centres, TORCS may offer additional advantages through enhanced three-dimensional intraoral visualisation and instrument dexterity, potentially enabling more precise dissection and tissue handling. However, further clinical studies are required to determine whether these technical advantages translate into superior communication-related outcomes. Specialised microsurgical robotic surgical instruments combined with a further development of haptic feedback might contribute to even better treatment outcomes and intraoperative safety. The implementation of artificial intelligence (AI) assistance, possibly combined with image-guided surgery, can aid in the recognition of important paediatric intraoral anatomical structures and in maintaining hemostatic control or improving surgical skills by data-driven feedback [9,40].

### 4.7. Study Limitations

This structured literature review has several limitations. Due to the limited availability of evidence, only six studies could be included. The majority of the included studies are preclinical, and none reported postoperative speech or velopharyngeal functional outcomes. In addition, evidence regarding otological outcomes was limited to a single observational study. The studies exhibit considerable heterogeneity in models, systems, and procedures. Both primary palatoplasty and secondary velopharyngeal procedures were included. As these procedures have different objectives, anatomical challenges, and expected functional outcomes, direct comparison between studies is limited and should be considered when interpreting the findings of this review. Finally, there is likely a degree of publication bias favouring positive feasibility findings, but no formal quality assessment has been performed.

## 5. Conclusions

Transoral robotic cleft surgery (TORCS) appears technically feasible and offers potential advantages in intraoral visualisation and surgeon ergonomics. However, current evidence remains limited and is predominantly preclinical. Preliminary clinical evidence suggests that TORCS may improve recovery of middle ear function after cleft palate repair; however, the available data remain limited. No conclusions can currently be drawn regarding speech outcomes. Further prospective clinical studies are needed to evaluate communication-related outcomes and to define the role of TORCS in cleft care.

## Figures and Tables

**Table 1:** Study characteristics.

Author	Year of Publication	Country	Type of Study	Type of Model Used	Amount of Patients/ Simulations	Controls	Follow-Up	Surgical Technique	Surgery Type	Robotic System	Tools Used	Sought-After Outcome Measures
Smartt et al. [24]	2013	United States of America	Preclinical	Cadaver	3 simulations	0	N/A	Posterior pharyngeal flap	Secondary/VPI procedure/pharyngoplasty	The Da Vinci S Surgical system	30-degree camera, two robotic surgical instruments	Technical: feasibility of robotic-enhanced soft palate reconstruction
Khan et al. [25]	2016	United Kingdom	Preclinical	Model + cadaver	2 simulations	0	N/A	Hynes pharyngoplasty	Secondary/VPI procedure/pharyngoplasty	The Da Vinci Si Surgical system	0-degree camera, two robotic (8 mm + 5 mm) surgical instruments	Technical: feasibility of robotic-enhanced cleft palate reconstruction
Nadjmi [29]	2016	Belgium	Preclinical + clinical	Cadaver + living cleft patients: 2 with BLCP, 2 with UCLP, 5 with CP, 1 with SMCP, age range: 9–10 months	1 simulation, 10 patients	30, manual, age range: 9–12 months	8 + −1 months	Modified Furlow palatoplasty	Primary palatoplasty	The Da Vinci Surgical system	30-degree camera, two robotic (8 mm + 5 mm) surgical instruments	Technical + clinical: feasibility of robotic-enhanced soft palate reconstruction
Podolsky et al. [26]	2016	Canada	Preclinical	Model	2 simulations	0	N/A	Von Langenbeck palatoplasty	Primary palatoplasty	The Da Vinci Si + Xi Surgical system	30-degree camera, two to three robotic (8 mm + 5 mm) surgical instruments	Technical: feasibility of robotic-enhanced cleft palate reconstruction
Téblick et al. [28]	2023	Belgium	Clinical	Living cleft patients: 7 with BCLP, 13 with UCLP, 9 with CP/CSP, age range: 9–10 months	29 patients	23, manual, 3 with BCLP, 10 with UCLP, 10 with CP/CSP, age range: 9–10 months	24 months	Furlow palatoplasty	Primary palatoplasty	The Da Vinci Surgical system	30-degree camera, two robotic surgical instruments	Clinical: speed of Eustachian tube function recovery by robotic-enhanced vs. manual cleft palate repair
Maguire et al. [27]	2025	Canada	Preclinical	Model	1 simulation	0	N/A	Two-flap palatoplasty	Primary palatoplasty	The Da Vinci Si + Xi Surgical system	Camera (not further specified), two robotic (3 mm + 8 mm) surgical instruments	Technical: feasibility of robotic-enhanced cleft palate reconstruction using 3 mm tools

**Table 2:** Summary of methodological quality assessment using the IDEAL framework.

Author	IDEAL Stage	Methodological Quality Regarding IDEAL Stage	Overall Risk of Bias	Certainty of Evidence for Clinical Application
Smartt et al. [24]	Stage 1 (Idea)	Good	High	Very Low
Khan et al. [25]	Stage 1 (Idea)	Good	High	Very Low
Podolsky et al. [26]	Stage 0 (Innovation)	Good	Moderate to High	Very Low
Nadjmi [29]	Stage 2a (Development)	Good	High	Low
Maguire et al. [27]	Stage 0 (Innovation)	Good	High	Very Low

## Data Availability

No data are available as no data were generated or analysed in this review.

## Supplementary Materials

The following supporting information can be downloaded at: https://www.mdpi.com/article/10.3390/s26134308/s1. Supplemental Figure S1: PRISMA flow diagram. Supplemental Table S1: Search planning form PubMed database. Supplemental Table S2: Search planning form Embase. Supplemental Table S3: Search planning form Cochrane Library. Supplemental Material S1: An overview of included and excluded publications. Supplemental Material S2: Risk of bias assessment.
