# Supplementary material for: Transoral Robotic Cleft Palate Surgery: Communication-Related Outcomes and Feasibility
Source: Sensors (Basel). 2026 Jul 7;26(13):4308. doi: 10.3390/s26134308 (PMC13364295; doi:10.3390/s26134308)
Supplement: Supplementary file 1 [file sensors-26-04308-s001.zip › sensors-4331713-supplementary.pdf]

Supplemental Figure S1: PRISMA flow diagram

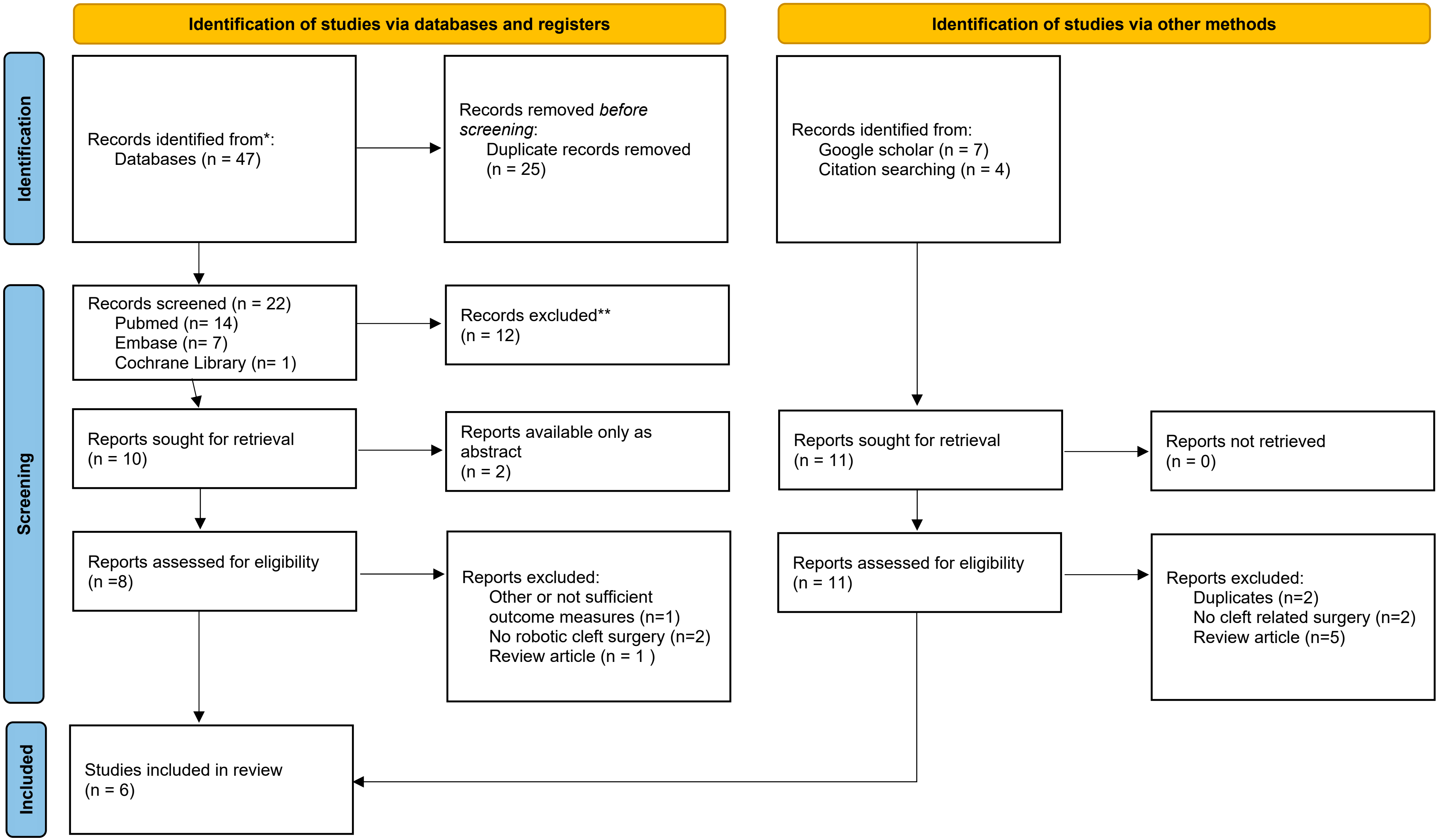

Supplemental Table S1: Search planning from PubMed database

**Date of search:**	19-01-2026								
**Research question:**	Is robotic-assisted cleft palate surgery able to improve communication more than manual cleft palate surgery?								
**Synonyms / different spellings / Alternative- / Controlled keywords**	**Concept 1**	**AND**	**Concept 2**	**AND**	**Concept 3**	**AND**	**Concept 4**	**AND**	**Concept 5**
	Robotic-assisted cleft palate surgery		Cleft palate		Velopharyngeal insufficiency associated speech difficulties		Audiological disease		Manual cleft palate surgery
	**Search terms**		**Search terms**		**Search terms**		**Search terms**		**Search terms**
	Robotic assisted cleft palate surgery		Cleft Palate (Mesh)		Velopharyngeal Insufficiency (Mesh)		Otitis Media with Effusion (Mesh)		Cleft Palate/surgery (Mesh)
	**OR**		**OR**		**OR**		**OR**		**OR**
	Robotic cleft palate surgery		CP/L		Speech Sound Disorder (Mesh)		Otitis Media (Mesh)		Palate, Hard/surgery (Mesh)
	**OR**		**OR**		**OR**		**OR**		**OR**
	robotic cleft surgery		CPO		Speech (Mesh)		Otitis Media, Suppurative (Mesh)		Palate, Soft/surgery (Mesh)
	**OR**		**OR**		**OR**		**OR**		**OR**
	robotic assisted cleft surgery		"cleft palat*"		Speech Intelligibility (Mesh)		Hearing loss (Mesh)		Cleft surgery
	**OR**		**OR**		**OR**		**OR**		**OR**
	"Robotic Surgical Procedures"[Mesh]		Cleft Soft Palate (Supplementary concept)		Speech Disorders (Mesh)		Hearing loss, Conductive (Mesh)		"Cleft palat* repair*"
	**OR**		**OR**		**OR**		**OR**		**OR**
	Robotic		Cleft hard palate		Speech Acoustics (Mesh)		Ear Diseases (Mesh)		Surgical cleft palate repair
	**OR**		**OR**		**OR**		**OR**		**OR**
	"Robotic assisted surgery"		Cleft soft palate		Velopharyngeal insufficiency associated speech difficulties		Otological disease		Cleft palate surgery
	**OR**		**OR**		**OR**		**OR**		**OR**

	"Robotic surgery"		Cleft palate only		"Velopharyn* in*"		"Otologic* dis*"		"Surgical Flaps"[Mesh]
	**OR**		**OR**		**OR**		**OR**		**OR**
	Robot		Cleft lip palate		"Velopharyn* dys*"		"Otiti* medi*"		posterior pharyngeal wall augmentation
	**OR**				**OR**		**OR**		**OR**
	"Robot-assisted"				Palatal function		Hearing loss		buccinator myomucosal flap
	**OR**				**OR**		**OR**		**OR**
	"Transoral robotic"						Conductive hearing loss		sphincter pharyngoplasty
	**OR**				"Velopharyngeal Sphincter"[Mesh]				**OR**
	"Transoral robotic surgery"				**OR**				Furlow double-opposing Z-plasty
					"Communication"[Mesh]				**OR**
					**OR**				posterior pharyngeal flap
					Communication Disorders [Mesh]				**OR**
					**OR**				Manual cleft palate surgery
					nasal turbulence				**OR**
					**OR**				"Manual cleft palat* surgery"
					hyponasality				**OR**
					**OR**				Palatoplasty
					hypernasality				**OR**
					**OR**				"Palat* surgery"
					hypernas*				**OR**
					**OR**				"Cleft palat* surgery"
					hyponas*				**OR**
					**OR**				"Cleft palat* repair"
					Communication disorders				
					**OR**				
					Communication				

			**OR**		
			"Nasal resonanc*"		
			**OR**		
			"Hypernasal* speech"		
			**OR**		
			"Nasal air leakage"		
			**OR**		
			"Nasal air escape"		
			**OR**		
			VPI		
			**OR**		
			Nasal emission		
			**OR**		
			Rhinolalia aperta		
	**Search string**	**Search string**	**Search string**	**Search string**	**Search string**
	((((((((((("Robotic Surgical Procedures"[Mesh]) OR ("robot assisted cleft palate surgery")) OR (robotic assisted cleft palate surgery)) OR (robotic cleft palate surgery)) OR (robotic cleft surgery)) OR (robotic assisted cleft surgery)) OR (robotic)) OR (robotic assisted surgery)) OR (robotic surgery)) OR (robot)) OR (robot assisted)) OR (transoral robotic)) OR (transoral robotic surgery)	(((((((("Cleft Palate"[Mesh]) OR (cleft palate)) OR (cleft soft palate)) OR (cleft hard palate)) OR ("Cleft Soft Palate" [Supplementary Concept])) OR (CP/L)) OR (Cleft palate only)) OR (cleft lip palate)) OR (CPO)) OR ("cleft palat*")	((((((((((((((((((velopharyngeal insufficiency associated speech difficulties) OR ((("Velopharyngeal Insufficiency"[Mesh]) OR "Speech Sound Disorder"[Mesh]) OR ("Speech"[Mesh] OR "Speech Disorders"[Mesh] OR "Speech Intelligibility"[Mesh] OR "Speech Acoustics"[Mesh]))) OR ("velopharyn* in*")) OR ("velopharyn* dys*")) OR (palatal function)) OR (("Velopharyngeal Sphincter"[Mesh]) OR ("Communication"[Mesh] OR "Communication Disorders"[Mesh]))) OR (nasal turbulence)) OR (nasal emission)) OR (hypernasality)) OR (hypernas*)) OR (hyponasality)) OR (hyponas*)) OR (communication disorders))	((((((("Otitis Media"[Mesh] OR "Otitis Media, Suppurative"[Mesh] OR "Otitis Media with Effusion"[Mesh]) OR ("Hearing Loss"[Mesh] OR "Hearing Loss, Conductive"[Mesh])) OR "Ear Diseases"[Mesh]) OR (otological disease)) OR ("otologic* dis*")) OR ("otiti* medi*")) OR (hearing loss)) OR (conductive hearing loss)	((((((((((((("Cleft Palate/surgery"[Mesh]) OR "Palate, Hard/surgery"[Mesh]) OR "Palate, Soft/surgery"[Mesh]) OR (cleft surgery)) OR (manual cleft palate surgery)) OR ("manual cleft palat* surgery")) OR (palatoplasty)) OR ("palat* surgery")) OR ("cleft palat* surgery")) OR ("cleft palat* repair")) OR (posterior pharyngeal flap)) OR (Furlow double-opposing Z-plasty)) OR (sphincter pharyngoplasty)) OR (buccinator myomucosal flap)) OR (Posterior

			OR (communication)) OR ("nasal resonanc*")) OR ("hypernasal* speech")) OR ("nasal air leakage")) OR ("nasal air escape")) OR (VPI)) OR (rhinolalia aperta)		pharyngeal wall augmentation)
	**Number of results**	**Number of results**	**Number of results**	**Number of results**	**Number of results**
	126,344	38,382	1,023,159	232,631	31,315
**Complete search string**	((((((((((((((((("Cleft Palate/surgery"[Mesh]) OR "Palate, Hard/surgery"[Mesh]) OR "Palate, Soft/surgery"[Mesh]) OR (cleft surgery)) OR (manual cleft palate surgery)) OR ("manual cleft palat* surgery")) OR (palatoplasty)) OR ("palat* surgery")) OR ("cleft palat* surgery")) OR ("cleft palat* repair")) OR (posterior pharyngeal flap)) OR (Furlow double-opposing Z-plasty)) OR (sphincter pharyngoplasty)) OR (buccinator myomucosal flap)) OR (Posterior pharyngeal wall augmentation)) AND (((((((("Otitis Media"[Mesh] OR "Otitis Media, Suppurative"[Mesh] OR "Otitis Media with Effusion"[Mesh]) OR ("Hearing Loss"[Mesh] OR "Hearing Loss, Conductive"[Mesh])) OR "Ear Diseases"[Mesh]) OR (otological disease)) OR ("otologic* dis*")) OR ("otiti* medi*")) OR (hearing loss)) OR (conductive hearing loss))) AND (((((((((((((((((((velopharyngeal insufficiency associated speech difficulties) OR ((("Velopharyngeal Insufficiency"[Mesh]) OR "Speech Sound Disorder"[Mesh]) OR ("Speech"[Mesh] OR "Speech Disorders"[Mesh] OR "Speech Intelligibility"[Mesh] OR "Speech Acoustics"[Mesh]))) OR ("velopharyn* in*")) OR ("velopharyn* dys*")) OR (palatal function)) OR (("Velopharyngeal Sphincter"[Mesh]) OR ("Communication"[Mesh] OR "Communication Disorders"[Mesh]))) OR (nasal turbulence)) OR (nasal emission)) OR (hypernasality)) OR (hypernas*)) OR (hyponasality)) OR (hyponas*)) OR (communication disorders)) OR (communication)) OR ("nasal resonanc*")) OR ("hypernasal* speech")) OR ("nasal air leakage")) OR ("nasal air escape")) OR (VPI)) OR (rhinolalia aperta))) AND (((((((((("Cleft Palate"[Mesh]) OR (cleft palate)) OR (cleft soft palate)) OR (cleft hard palate)) OR ("Cleft Soft Palate" [Supplementary Concept])) OR (CP/L)) OR (Cleft palate only)) OR (cleft lip palate)) OR (CPO)) OR ("cleft palat*"))) AND ((((((((((((("Robotic Surgical Procedures"[Mesh]) OR ("robot assisted cleft palate surgery")) OR (robotic assisted cleft palate surgery)) OR (robotic cleft palate surgery)) OR (robotic cleft surgery)) OR (robotic assisted cleft surgery)) OR (robotic)) OR (robotic assisted surgery)) OR (robotic surgery)) OR (robot)) OR (robot assisted)) OR (transoral robotic)) OR (transoral robotic surgery))				
**Alternative search string 1**	(((((((((((((("Robotic Surgical Procedures"[Mesh]) OR ("robot assisted cleft palate surgery")) OR (robotic assisted cleft palate surgery)) OR (robotic cleft palate surgery)) OR (robotic cleft surgery)) OR (robotic assisted cleft surgery)) OR (robotic)) OR (robotic assisted surgery)) OR (robotic surgery)) OR (robot)) OR (robot assisted)) OR (transoral robotic)) OR (transoral robotic surgery)) AND (((((((((("Cleft Palate"[Mesh]) OR (cleft palate)) OR (cleft soft palate)) OR (cleft hard palate)) OR ("Cleft Soft Palate" [Supplementary Concept])) OR (CP/L)) OR (Cleft palate only)) OR (cleft lip palate)) OR (CPO)) OR ("cleft palat*"))) AND (((((((((((((((((((velopharyngeal insufficiency associated speech difficulties) OR ((("Velopharyngeal Insufficiency"[Mesh]) OR "Speech Sound Disorder"[Mesh]) OR ("Speech"[Mesh] OR "Speech Disorders"[Mesh] OR "Speech Intelligibility"[Mesh] OR "Speech Acoustics"[Mesh]))) OR ("velopharyn* in*")) OR ("velopharyn* dys*")) OR (palatal function)) OR (("Velopharyngeal Sphincter"[Mesh]) OR ("Communication"[Mesh] OR "Communication Disorders"[Mesh]))) OR (nasal turbulence)) OR (nasal emission)) OR (hypernasality)) OR (hypernas*)) OR (hyponasality)) OR (hyponas*)) OR (communication disorders)) OR (communication)) OR ("nasal resonanc*")) OR ("hypernasal* speech")) OR ("nasal air leakage")) OR ("nasal air escape")) OR (VPI)) OR (rhinolalia aperta))) AND ((((((((((((((("Cleft Palate/surgery"[Mesh]) OR "Palate, Hard/surgery"[Mesh]) OR "Palate, Soft/surgery"[Mesh]) OR (cleft surgery)) OR (manual cleft palate surgery)) OR ("manual cleft palat* surgery")) OR (palatoplasty)) OR ("palat* surgery")) OR ("cleft palat* surgery")) OR ("cleft palat* repair")) OR (posterior pharyngeal flap)) OR (Furlow double-opposing Z-plasty)) OR (sphincter pharyngoplasty)) OR (buccinator myomucosal flap)) OR (Posterior pharyngeal wall augmentation))				
**Alternative search string 2**	((((((((((((((((("Cleft Palate/surgery"[Mesh]) OR "Palate, Hard/surgery"[Mesh]) OR "Palate, Soft/surgery"[Mesh]) OR (cleft surgery)) OR (manual cleft palate surgery)) OR ("manual cleft palat* surgery")) OR (palatoplasty)) OR ("palat* surgery")) OR ("cleft palat* surgery")) OR ("cleft palat* repair")) OR (posterior pharyngeal flap)) OR (Furlow double-opposing Z-plasty)) OR (sphincter pharyngoplasty)) OR (buccinator myomucosal flap)) OR (Posterior pharyngeal wall augmentation)) AND (((((((("Otitis Media"[Mesh] OR "Otitis Media, Suppurative"[Mesh] OR "Otitis Media with Effusion"[Mesh]) OR ("Hearing Loss"[Mesh] OR "Hearing Loss, Conductive"[Mesh])) OR "Ear Diseases"[Mesh]) OR (otological disease)) OR ("otologic* dis*")) OR ("otiti* medi*")) OR (hearing loss)) OR (conductive hearing loss))) AND (((((((((("Cleft Palate"[Mesh]) OR (cleft palate)) OR (cleft soft palate)) OR (cleft hard palate)) OR ("Cleft Soft Palate" [Supplementary Concept])) OR (CP/L)) OR (Cleft palate only)) OR (cleft lip palate)) OR (CPO)) OR ("cleft palat*"))) AND				

	((((((((((((("Robotic Surgical Procedures"[Mesh]) OR ("robot assisted cleft palate surgery")) OR (robotic assisted cleft palate surgery)) OR (robotic cleft palate surgery)) OR (robotic cleft surgery)) OR (robotic assisted cleft surgery)) OR (robotic)) OR (robotic assisted surgery)) OR (robotic surgery)) OR (robot)) OR (robot assisted)) OR (transoral robotic)) OR (transoral robotic surgery))
**Alternative search string 3**	(((((((((((((("Robotic Surgical Procedures"[Mesh]) OR ("robot assisted cleft palate surgery")) OR (robotic assisted cleft palate surgery)) OR (robotic cleft palate surgery)) OR (robotic cleft surgery)) OR (robotic assisted cleft surgery)) OR (robotic)) OR (robotic assisted surgery)) OR (robotic surgery)) OR (robot)) OR (robot assisted)) OR (transoral robotic)) OR (transoral robotic surgery)) AND (((((((((("Cleft Palate"[Mesh]) OR (cleft palate)) OR (cleft soft palate)) OR (cleft hard palate)) OR ("Cleft Soft Palate" [Supplementary Concept])) OR (CP/L)) OR (Cleft palate only)) OR (cleft lip palate)) OR (CPO)) OR ("cleft palat*"))) AND ((((((((((((((((((((velopharyngeal insufficiency associated speech difficulties) OR ((("Velopharyngeal Insufficiency"[Mesh]) OR "Speech Sound Disorder"[Mesh]) OR ("Speech"[Mesh] OR "Speech Disorders"[Mesh] OR "Speech Intelligibility"[Mesh] OR "Speech Acoustics"[Mesh]))) OR ("velopharyn* in*")) OR ("velopharyn* dys*")) OR (palatal function)) OR (("Velopharyngeal Sphincter"[Mesh]) OR ("Communication"[Mesh] OR "Communication Disorders"[Mesh]))) OR (nasal turbulence)) OR (nasal emission)) OR (hypernasality)) OR (hypernas*)) OR (hyponasality)) OR (hyponas*)) OR (communication disorders)) OR (communication)) OR ("nasal resonanc*")) OR ("hypernasal* speech")) OR ("nasal air leakage")) OR ("nasal air escape")) OR (VPI)) OR (rhinolalia aperta))
**Alternative search string 4**	(((((((((((((("Robotic Surgical Procedures"[Mesh]) OR ("robot assisted cleft palate surgery")) OR (robotic assisted cleft palate surgery)) OR (robotic cleft palate surgery)) OR (robotic cleft surgery)) OR (robotic assisted cleft surgery)) OR (robotic)) OR (robotic assisted surgery)) OR (robotic surgery)) OR (robot)) OR (robot assisted)) OR (transoral robotic)) OR (transoral robotic surgery)) AND (((((((((("Cleft Palate"[Mesh]) OR (cleft palate)) OR (cleft soft palate)) OR (cleft hard palate)) OR ("Cleft Soft Palate" [Supplementary Concept])) OR (CP/L)) OR (Cleft palate only)) OR (cleft lip palate)) OR (CPO)) OR ("cleft palat*"))) AND (((((((("Otitis Media"[Mesh] OR "Otitis Media, Suppurative"[Mesh] OR "Otitis Media with Effusion"[Mesh]) OR ("Hearing Loss"[Mesh] OR "Hearing Loss, Conductive"[Mesh])) OR "Ear Diseases"[Mesh]) OR (otological disease)) OR ("otologic* dis*")) OR ("otiti* medi*")) OR (hearing loss)) OR (conductive hearing loss))
**Total number of results**	15
**Source/Database:**	PubMed
	**Search limits**
**Study type:**	
**Age range:**	
**Date range:**	
**Language:**	Dutch, English
**Other:**	

Supplemental Table S2: Search planning from Embase

**Date of search:**									19-01-2026
**Research question:**	Is robotic-assisted cleft palate surgery able to improve communication more than manual cleft palate surgery?								
**Synonyms / different spellings / Alternative- / Controlled keywords**	**Concept 1**	**AND**	**Concept 2**	**AND**	**Concept 3**	**AND**	**Concept 4**	**AND**	**Concept 5**
	Robotic-assisted cleft palate surgery		Cleft palate		Velopharyngeal insufficiency associated speech difficulties		Audiological disease		Manual cleft palate surgery
	**Search terms**		**Search terms**		**Search terms**		**Search terms**		**Search terms**
	robotic assisted cleft palate surgery.mp.		exp cleft palate/		exp palatopharyngeal incompetence/		exp otitis media/		exp cleft palate/su [Surgery]
	**OR**		**OR**		**OR**		**OR**		**OR**
	exp robot assisted surgery/		exp cleft soft palate/		velopharyngeal insufficiency.mp.		otitis media.mp.		exp cleft hard palate/dm [Disease Management]
	**OR**		**OR**		**OR**		**OR**		**OR**
	exp robotics/		cleft hard palate/		exp speech/		otitis media with effusion.mp.		exp cleft soft palate/su [Surgery]
	**OR**		**OR**		**OR**		**OR**		**OR**
	robot assisted surgery.mp.		cleft palate only.mp.		exp speech disorder/		exp secretory otitis media/		exp cleft lip palate/su [Surgery]
	**OR**		**OR**		**OR**		**OR**		**OR**
	robotic cleft palate surgery.mp.		cleft lip palate.mp.		speech sound disorder.mp.		exp suppurative otitis media/		cleft surgery.mp.
	**OR**		**OR**		**OR**		**OR**		**OR**
	exp transoral robotic surgery/		exp cleft lip palate/		exp speech sound disorder/		otitis media suppurative.mp.		cleft palat* repair*.mp.
	**OR**		**OR**		**OR**		**OR**		**OR**
	robotic cleft surgery.mp.		cleft soft palate.mp.		exp speech intelligibility/		exp hearing impairment/		surgical cleft palate repair.mp.
	**OR**		**OR**		**OR**		**OR**		**OR**
	transoral robotic surgery.mp.		cleft palate.mp.		speech intelligibility.mp.		hearing loss.mp.		cleft palate surgery.mp.

	**OR**		**OR**		**OR**		**OR**		**OR**
	transoral robotic.mp.		cleft hard palate.mp.		speech acoustics.mp.		conduction deafness/		exp palatoplasty/
	**OR**		**OR**		**OR**		**OR**		**OR**
	robotic assisted cleft surgery.mp.		exp cleft hard palate/		velopharyngeal insufficiency associated speech difficulties.mp.		conductive hearing loss.mp.		palatoplasty.mp.
	**OR**		**OR**		**OR**		**OR**		**OR**
	robotical surgical procedures.mp.		CPO.mp.		velopharyn* dys*.mp.		ear diseases.mp.		surgical flaps.mp.
	**OR**		**OR**		**OR**		**OR**		**OR**
	robotic surgery.mp.		CPL.mp.		palatal function.mp.		exp ear disease/		surgical flaps/
	**OR**		**OR**		**OR**		**OR**		**OR**
	exp robot-assisted procedure/		cleft palat*.mp.		exp velopharyngeal sphincter/		otological disease.mp.		posterior pharyngeal wall augmentation.mp.
	**OR**				**OR**		**OR**		**OR**
	robot.mp.				velopharyngeal sphincter.mp.		otologic* dis*.mp.		buccinator myomucosal flap.mp.
	**OR**				**OR**		**OR**		**OR**
	exp robot/				exp interpersonal communication/		otiti* medi*.mp.		sphincter pharyngoplasty.mp.
	**OR**				**OR**				**OR**
	robot assisted.mp.				communication.mp.				exp velopharyngeal sphincter/su [Surgery]
					**OR**				**OR**
					exp communication disorder/				exp palatopharyngeal incompetence/su [Surgery]
					**OR**				**OR**
					communication disorders.mp.				furlow double-opposing Z-plasty.mp.
					**OR**				**OR**
					nasal turbulence.mp.				exp Z plasty/

					**OR**				**OR**
					exp nose airflow/				posterior pharyngeal flap.mp.
					**OR**				**OR**
					nasal emission.mp.				manual cleft palate surgery.mp.
					**OR**				**OR**
					exp hypernasality/				palat* surgery.mp.
					**OR**				**OR**
					hypernasality.mp.				cleft palat* surgery.mp.
					**OR**				**OR**
					exp nasal speech/				cleft palat* repair.mp.
					**OR**				**OR**
					hyponasality.mp.				manual surgery.mp.
					**OR**				
					hypernas*.mp.				
					**OR**				
					hyponas*.mp.				
					**OR**				
					nasal reson*.mp.				
					**OR**				
					hypernasal* speech.mp.				
					**OR**				
					nasal air leakage.mp.				
					**OR**				
					nasal air escape.mp.				
					**OR**				
					VPI.mp.				

					**OR**				
					rhinolalia aperta.mp.				
	**Search string**		**Search string**		**Search string**		**Search string**		**Search string**
	(robot assisted.mp.) OR (exp robot/) OR (robot.mp.) OR (exp robot-assisted procedure/) OR (robotic surgery.mp.) OR (robotical surgical procedures.mp.) OR (robotic assisted cleft surgery.mp.) OR (transoral robotic.mp.) OR (transoral robotic surgery.mp.) OR (robotic cleft surgery.mp.) OR (exp transoral robotic surgery/) OR (robotic cleft palate surgery.mp.) OR (exp robotics/) OR (robot assisted surgery.mp.) OR (robotic assisted cleft palate surgery.mp.) OR (exp robot assisted surgery/)		(cleft palat*.mp.) OR (CPL.mp.) OR (CPO.mp.) OR (cleft hard palate.mp.) OR (exp cleft hard palate/) OR (cleft palate.mp.) OR (cleft soft palate.mp.) OR (cleft lip palate.mp.) OR (exp cleft lip palate/) OR (cleft palate only.mp.) OR (cleft hard palate/) OR (exp cleft soft palate/) OR (exp cleft palate/)		(rhinolalia aperta.mp.) OR (VPI.mp.) OR (nasal air escape.mp.) OR (nasal air leakage.mp.) OR (hypernasal* speech.mp.) OR (nasal reson*.mp.) OR (hyponas*.mp.) OR (hypernas*.mp.) OR (hyponasality.mp.) OR (hypernasality.mp.) OR (exp nasal speech/) OR (exp hypernasality/) OR (nasal emission.mp.) OR (exp nose airflow/) OR (nasal turbulence.mp.) OR (communication disorders.mp.) OR (exp communication disorder/) OR (communication.mp.) OR (exp interpersonal communication/) OR (velopharyngeal sphincter.mp.) OR (exp velopharyngeal sphincter/) OR (palatal function.mp.) OR (velopharyn* dys*.mp.) OR (velopharyngeal insufficiency associated speech difficulties.mp.) OR (speech acoustics.mp.) OR (exp speech intelligibility/) OR (speech intelligibility.mp.) OR (exp speech sound disorder/) OR (speech sound disorder.mp.) OR (exp speech disorder/) OR (exp speech/) OR (velopharyngeal insufficiency.mp.) OR (exp		(otiti* medi*.mp.) OR (otologic* dis*.mp.) OR (otological disease.mp.) OR (exp ear disease/) OR (ear diseases.mp.) OR (conduction deafness/) OR (conductive hearing loss.mp.) OR (exp hearing impairment/) OR (hearing loss.mp.) OR (exp suppurative otitis media/) OR (otitis media suppurative.mp.) OR (otitis media with effusion.mp.) OR (exp secretory otitis media/) OR (exp otitis media/) OR (otitis media.mp.)		(manual surgery.mp.) OR (cleft palat* repair.mp.) OR (cleft palat* surgery.mp.) OR (palat* surgery.mp.) OR (manual cleft palate surgery.mp.) OR (posterior pharyngeal flap.mp.) OR (exp Z plasty/) OR (furlow double-opposing Z-plasty.mp.) OR (exp palatopharyngeal incompetence/su [Surgery]) OR (exp velopharyngeal sphincter/su [Surgery]) OR (sphincter pharyngoplasty.mp.) OR (buccinator myomucosal flap.mp.) OR (posterior pharyngeal wall augmentation.mp.) OR (surgical flaps/) OR (surgical flaps.mp.) OR (palatoplasty.mp.) OR (exp palatoplasty/) OR (cleft palate surgery.mp.) OR (surgical cleft palate repair.mp.) OR (cleft palat* repair*.mp.) OR (cleft surgery.mp.) OR (exp cleft lip palate/su [Surgery]) OR (exp cleft soft palate/su [Surgery]) OR (exp cleft hard palate/dm [Disease Management]) OR (exp cleft palate/su [Surgery])

					palatopharyngeal incompetence/)				
	**Number of results**		**Number of results**		**Number of results**		**Number of results**		**Number of results**
	122593		40969		1,520,114		295839		24347
**Complete search string**	((robot assisted.mp.) OR (exp robot/) OR (robot.mp.) OR (exp robot-assisted procedure/) OR (robotic surgery.mp.) OR (robotical surgical procedures.mp.) OR (robotic assisted cleft surgery.mp.) OR (transoral robotic.mp.) OR (transoral robotic surgery.mp.) OR (robotic cleft surgery.mp.) OR (exp transoral robotic surgery/) OR (robotic cleft palate surgery.mp.) OR (exp robotics/) OR (robot assisted surgery.mp.) OR (robotic assisted cleft palate surgery.mp.) OR (exp robot assisted surgery/)) AND ((cleft palat*.mp.) OR (CPL.mp.) OR (CPO.mp.) OR (cleft hard palate.mp.) OR (exp cleft hard palate/) OR (cleft palate.mp.) OR (cleft soft palate.mp.) OR (cleft lip palate.mp.) OR (exp cleft lip palate/) OR (cleft palate only.mp.) OR (cleft hard palate/) OR (exp cleft soft palate/) OR (exp cleft palate/)) AND ((rhinolalia aperta.mp.) OR (VPI.mp.) OR (nasal air escape.mp.) OR (nasal air leakage.mp.) OR (hypernasal* speech.mp.) OR (nasal reson*.mp.) OR (hyponas*.mp.) OR (hypernas*.mp.) OR (hyponasality.mp.) OR (hypernasality.mp.) OR (exp nasal speech/) OR (exp hypernasality/) OR (nasal emission.mp.) OR (exp nose airflow/) OR (nasal turbulence.mp.) OR (communication disorders.mp.) OR (exp communication disorder/) OR (communication.mp.) OR (exp interpersonal communication/) OR (velopharyngeal sphincter.mp.) OR (exp velopharyngeal sphincter/) OR (palatal function.mp.) OR (velopharyn* dys*.mp.) OR (velopharyngeal insufficiency associated speech difficulties.mp.) OR (speech acoustics.mp.) OR (exp speech intelligibility/) OR (speech intelligibility.mp.) OR (exp speech sound disorder/) OR (speech sound disorder.mp.) OR (exp speech disorder/) OR (exp speech/) OR (velopharyngeal insufficiency.mp.) OR (exp palatopharyngeal incompetence/)) AND ((otiti* medi*.mp.) OR (otologic* dis*.mp.) OR (otological disease.mp.) OR (exp ear disease/) OR (ear diseases.mp.) OR (conduction deafness/) OR (conductive hearing loss.mp.) OR (exp hearing impairment/) OR (hearing loss.mp.) OR (exp suppurative otitis media/) OR (otitis media suppurative.mp.) OR (otitis media with effusion.mp.) OR (exp secretory otitis media/) OR (exp otitis media/) OR (otitis media.mp.)) AND ((manual surgery.mp.) OR (cleft palat* repair.mp.) OR (cleft palat* surgery.mp.) OR (palat* surgery.mp.) OR (manual cleft palate surgery.mp.) OR (posterior pharyngeal flap.mp.) OR (exp Z plasty/) OR (furlow double-opposing Z-plasty.mp.) OR (exp palatopharyngeal incompetence/su [Surgery]) OR (exp velopharyngeal sphincter/su [Surgery]) OR (sphincter pharyngoplasty.mp.) OR (buccinator myomucosal flap.mp.) OR (posterior pharyngeal wall augmentation.mp.) OR (surgical flaps/) OR (surgical flaps.mp.) OR (palatoplasty.mp.) OR (exp palatoplasty/) OR (cleft palate surgery.mp.) OR (surgical cleft palate repair.mp.) OR (cleft palat* repair*.mp.) OR (cleft surgery.mp.) OR (exp cleft lip palate/su [Surgery]) OR (exp cleft soft palate/su [Surgery]) OR (exp cleft hard palate/dm [Disease Management]) OR (exp cleft palate/su [Surgery]))								
**Alternative search string 1**	((robot assisted.mp.) OR (exp robot/) OR (robot.mp.) OR (exp robot-assisted procedure/) OR (robotic surgery.mp.) OR (robotical surgical procedures.mp.) OR (robotic assisted cleft surgery.mp.) OR (transoral robotic.mp.) OR (transoral robotic surgery.mp.) OR (robotic cleft surgery.mp.) OR (exp transoral robotic surgery/) OR (robotic cleft palate surgery.mp.) OR (exp robotics/) OR (robot assisted surgery.mp.) OR (robotic assisted cleft palate surgery.mp.) OR (exp robot assisted surgery/)) AND ((cleft palat*.mp.) OR (CPL.mp.) OR (CPO.mp.) OR (cleft hard palate.mp.) OR (exp cleft hard palate/) OR (cleft palate.mp.) OR (cleft soft palate.mp.) OR (cleft lip palate.mp.) OR (exp cleft lip palate/) OR (cleft palate only.mp.) OR (cleft hard palate/) OR (exp cleft soft palate/) OR (exp cleft palate/)) AND ((rhinolalia aperta.mp.) OR (VPI.mp.) OR (nasal air escape.mp.) OR (nasal air leakage.mp.) OR (hypernasal* speech.mp.) OR (nasal reson*.mp.) OR (hyponas*.mp.) OR (hypernas*.mp.) OR (hyponasality.mp.) OR (hypernasality.mp.) OR (exp nasal speech/) OR (exp hypernasality/) OR (nasal emission.mp.) OR (exp nose airflow/) OR (nasal turbulence.mp.) OR (communication disorders.mp.) OR (exp communication disorder/) OR (communication.mp.) OR (exp interpersonal communication/) OR (velopharyngeal sphincter.mp.) OR (exp velopharyngeal sphincter/) OR (palatal function.mp.) OR (velopharyn* dys*.mp.) OR (velopharyngeal insufficiency associated speech difficulties.mp.) OR (speech acoustics.mp.) OR (exp speech intelligibility/) OR (speech intelligibility.mp.) OR (exp speech sound disorder/) OR (speech sound disorder.mp.) OR (exp speech disorder/) OR (exp speech/) OR (velopharyngeal insufficiency.mp.) OR (exp palatopharyngeal incompetence/)) AND ((manual surgery.mp.) OR (cleft palat* repair.mp.) OR (cleft palat* surgery.mp.) OR (palat* surgery.mp.) OR (manual cleft palate surgery.mp.) OR (posterior pharyngeal flap.mp.) OR (exp Z plasty/) OR (furlow double-opposing Z-plasty.mp.) OR (exp palatopharyngeal incompetence/su [Surgery]) OR (exp velopharyngeal sphincter/su [Surgery]) OR (sphincter pharyngoplasty.mp.) OR (buccinator myomucosal flap.mp.) OR (posterior pharyngeal wall augmentation.mp.) OR (surgical flaps/) OR								

	(surgical flaps.mp.) OR (palatoplasty.mp.) OR (exp palatoplasty/) OR (cleft palate surgery.mp.) OR (surgical cleft palate repair.mp.) OR (cleft palat* repair*.mp.) OR (cleft surgery.mp.) OR (exp cleft lip palate/su [Surgery]) OR (exp cleft soft palate/su [Surgery]) OR (exp cleft hard palate/dm [Disease Management]) OR (exp cleft palate/su [Surgery]))
**Alternative search string 2**	((robot assisted.mp.) OR (exp robot/) OR (robot.mp.) OR (exp robot-assisted procedure/) OR (robotic surgery.mp.) OR (robotical surgical procedures.mp.) OR (robotic assisted cleft surgery.mp.) OR (transoral robotic.mp.) OR (transoral robotic surgery.mp.) OR (robotic cleft surgery.mp.) OR (exp transoral robotic surgery/) OR (robotic cleft palate surgery.mp.) OR (exp robotics/) OR (robot assisted surgery.mp.) OR (robotic assisted cleft palate surgery.mp.) OR (exp robot assisted surgery/)) AND ((cleft palat*.mp.) OR (CPL.mp.) OR (CPO.mp.) OR (cleft hard palate.mp.) OR (exp cleft hard palate/) OR (cleft palate.mp.) OR (cleft soft palate.mp.) OR (cleft lip palate.mp.) OR (exp cleft lip palate/) OR (cleft palate only.mp.) OR (cleft hard palate/) OR (exp cleft soft palate/) OR (exp cleft palate/)) AND ((otiti* medi*.mp.) OR (otologic* dis*.mp.) OR (otological disease.mp.) OR (exp ear disease/) OR (ear diseases.mp.) OR (conduction deafness/) OR (conductive hearing loss.mp.) OR (exp hearing impairment/) OR (hearing loss.mp.) OR (exp suppurative otitis media/) OR (otitis media suppurative.mp.) OR (otitis media with effusion.mp.) OR (exp secretory otitis media/) OR (exp otitis media/) OR (otitis media.mp.)) AND ((manual surgery.mp.) OR (cleft palat* repair.mp.) OR (cleft palat* surgery.mp.) OR (palat* surgery.mp.) OR (manual cleft palate surgery.mp.) OR (posterior pharyngeal flap.mp.) OR (exp Z plasty/) OR (furlow double-opposing Z-plasty.mp.) OR (exp palatopharyngeal incompetence/su [Surgery]) OR (exp velopharyngeal sphincter/su [Surgery]) OR (sphincter pharyngoplasty.mp.) OR (buccinator myomucosal flap.mp.) OR (posterior pharyngeal wall augmentation.mp.) OR (surgical flaps/) OR (surgical flaps.mp.) OR (palatoplasty.mp.) OR (exp palatoplasty/) OR (cleft palate surgery.mp.) OR (surgical cleft palate repair.mp.) OR (cleft palat* repair*.mp.) OR (cleft surgery.mp.) OR (exp cleft lip palate/su [Surgery]) OR (exp cleft soft palate/su [Surgery]) OR (exp cleft hard palate/dm [Disease Management]) OR (exp cleft palate/su [Surgery]))
**Alternative search string 3**	((robot assisted.mp.) OR (exp robot/) OR (robot.mp.) OR (exp robot-assisted procedure/) OR (robotic surgery.mp.) OR (robotical surgical procedures.mp.) OR (robotic assisted cleft surgery.mp.) OR (transoral robotic.mp.) OR (transoral robotic surgery.mp.) OR (robotic cleft surgery.mp.) OR (exp transoral robotic surgery/) OR (robotic cleft palate surgery.mp.) OR (exp robotics/) OR (robot assisted surgery.mp.) OR (robotic assisted cleft palate surgery.mp.) OR (exp robot assisted surgery/)) AND ((cleft palat*.mp.) OR (CPL.mp.) OR (CPO.mp.) OR (cleft hard palate.mp.) OR (exp cleft hard palate/) OR (cleft palate.mp.) OR (cleft soft palate.mp.) OR (cleft lip palate.mp.) OR (exp cleft lip palate/) OR (cleft palate only.mp.) OR (cleft hard palate/) OR (exp cleft soft palate/) OR (exp cleft palate/)) AND ((rhinolalia aperta.mp.) OR (VPI.mp.) OR (nasal air escape.mp.) OR (nasal air leakage.mp.) OR (hypernasal* speech.mp.) OR (nasal reson*.mp.) OR (hyponas*.mp.) OR (hypernas*.mp.) OR (hyponasality.mp.) OR (hypernasality.mp.) OR (exp nasal speech/) OR (exp hypernasality/) OR (nasal emission.mp.) OR (exp nose airflow/) OR (nasal turbulence.mp.) OR (communication disorders.mp.) OR (exp communication disorder/) OR (communication.mp.) OR (exp interpersonal communication/) OR (velopharyngeal sphincter.mp.) OR (exp velopharyngeal sphincter/) OR (palatal function.mp.) OR (velopharyn* dys*.mp.) OR (velopharyngeal insufficiency associated speech difficulties.mp.) OR (speech acoustics.mp.) OR (exp speech intelligibility/) OR (speech intelligibility.mp.) OR (exp speech sound disorder/) OR (speech sound disorder.mp.) OR (exp speech disorder/) OR (exp speech/) OR (velopharyngeal insufficiency.mp.) OR (exp palatopharyngeal incompetence/))
**Alternative search string 4**	((robot assisted.mp.) OR (exp robot/) OR (robot.mp.) OR (exp robot-assisted procedure/) OR (robotic surgery.mp.) OR (robotical surgical procedures.mp.) OR (robotic assisted cleft surgery.mp.) OR (transoral robotic.mp.) OR (transoral robotic surgery.mp.) OR (robotic cleft surgery.mp.) OR (exp transoral robotic surgery/) OR (robotic cleft palate surgery.mp.) OR (exp robotics/) OR (robot assisted surgery.mp.) OR (robotic assisted cleft palate surgery.mp.) OR (exp robot assisted surgery/)) AND ((cleft palat*.mp.) OR (CPL.mp.) OR (CPO.mp.) OR (cleft hard palate.mp.) OR (exp cleft hard palate/) OR (cleft palate.mp.) OR (cleft soft palate.mp.) OR (cleft lip palate.mp.) OR (exp cleft lip palate/) OR (cleft palate only.mp.) OR (cleft hard palate/) OR (exp cleft soft palate/) OR (exp cleft palate/)) AND ((otiti* medi*.mp.) OR (otologic* dis*.mp.) OR (otological disease.mp.) OR (exp ear disease/) OR (ear diseases.mp.) OR (conduction deafness/) OR (conductive hearing loss.mp.) OR (exp hearing impairment/) OR (hearing loss.mp.) OR (exp suppurative otitis media/) OR (otitis media suppurative.mp.) OR (otitis media with effusion.mp.) OR (exp secretory otitis media/) OR (exp otitis media/) OR (otitis media.mp.))
**Total number of results**	23
**Source/Database:**	Embase

Search limits	
**Study type:**	
**Age range:**	
**Date range:**	
**Language:**	Dutch, English
**Other:**	

Supplemental Table S3: Search planning from Cochrane Library

**Date of search:**	19-01-2026									
**Research question:**	Is robotic-assisted cleft palate surgery able to improve communication more than manual cleft palate surgery?									
**Synonyms / different spellings / Alternative- / Controlled keywords**	**Concept 1**	**AND**	**Concept 2**	**AND**	**Concept 3**	**AND**	**Concept 4**	**AND**	**Concept 5**
	Robotic-assisted cleft palate surgery		Cleft palate		Velopharyngeal insufficiency associated speech difficulties		Audiological disease		Manual cleft palate surgery
	**Search terms**		**Search terms**		**Search terms**		**Search terms**		**Search terms**
	Robotic assisted cleft palate surgery		Cleft Palate [Mesh]		Velopharyngeal Insufficiency [Mesh]		Otitis Media with Effusion [Mesh]		Cleft Palate surgery
	**OR**		**OR**		**OR**		**OR**		**OR**
	Robotic cleft palate surgery		Cleft Soft Palate		Speech Sound Disorder [Mesh]		Otitis Media [Mesh]		Cleft surgery
	**OR**		**OR**		**OR**		**OR**		**OR**
	robotic cleft surgery		Cleft Hard Palate		Speech [Mesh]		Otitis Media, Suppurative [Mesh]		Cleft palat* NEXT repair*
	**OR**		**OR**		**OR**		**OR**		**OR**
	robotic assisted cleft surgery		Cleft NEXT palat*		Speech Intelligibility [Mesh]		Hearing loss [Mesh]		Surgical cleft palate repair
	**OR**				**OR**		**OR**		**OR**
	Robotic Surgical Procedures [Mesh]				Speech Disorders		Hearing loss, Conductive [Mesh]		Surgical Flaps [Mesh]
	**OR**				**OR**		**OR**		**OR**
	Robotic				Speech Acoustics		Ear Diseases [Mesh]		Posterior pharyngeal wall augmentation
	**OR**				**OR**		**OR**		**OR**
	"Robotic assisted surgery"				Velopharyngeal insufficiency associated speech difficulties		Otological disease		Buccinator myomucosal flaps
	**OR**				**OR**		**OR**		**OR**
	"Robotic surgery"				Velopharyn* in*		Otologic* NEXT dis*		Sphincter pharyngoplasty

	**OR**				**OR**		**OR**		**OR**
	Robot				Velopharyn* dys*		Otiti* NEXT medi*		Furlow double-opposing Z-plasty
	**OR**				**OR**		**OR**		**OR**
	Robot-assisted				Palatal function		Hearing loss		Posterior pharyngeal flap
	**OR**				**OR**		**OR**		**OR**
	Transoral robotic				Velopharyngeal Sphincter [Mesh]		Conductive hearing loss		Palatoplasty
	**OR**								**OR**
	Transoral robotic surgery				**OR**				Palat* NEAR surgery
					Communication disorder [Mesh]				**OR**
					**OR**				Cleft NEXT palat* NEAR surgery
					Nasal emission				**OR**
					**OR**				Cleft NEXT palat* NEAR repair
					nasal turbulence				**OR**
					**OR**				Manual cleft palate surgery
					hyponasality				**OR**
					**OR**				Manual NEAR cleft NEXT palat* NEAR surgery
					hypernasality				
					**OR**				
					Articulation disorder [Mesh]				
					**OR**				
					Nasal NEXT resonanc*				
					**OR**				
					Hypernasal* NEXT speech				
					**OR**				

					Hypernasal*<br>**OR**<br>Nasal NEAR air NEAR leak<br>**OR**<br>Nasal NEAR air NEAR escape<br>**OR**<br>Nasal NEXT turbulence<br>**OR**<br>Nasal rustle<br>**OR**<br>VPI<br>**OR**<br>Rhinolalia aperta				
	**Search string**		**(Cleft Palate [Mesh]) OR (Cleft Soft Palate) OR (Cleft Hard Palate) OR (Cleft NEXT palat*)**		**Search string**		**Search string**		**Search string**
	(Robotic assisted cleft palate surgery) OR (Robotic cleft palate surgery) OR (robotic cleft surgery) OR (robotic assisted cleft surgery) OR (Robotic Surgical Procedures [Mesh]) OR (Robotic) OR (Robotic assisted surgery) OR (Robotic surgery) OR (Robot) OR (Robot-assisted) OR (Transoral		(Cleft Palate [Mesh]) OR (Cleft Soft Palate) OR (Cleft Hard Palate) OR (Cleft NEXT palat*)		(Velopharyngeal Insufficiency [Mesh]) OR (Speech Sound Disorder [Mesh]) OR (Speech [Mesh]) OR (Speech Intelligibility [Mesh]) OR (Speech Disorders) OR (Speech Acoustics) OR (Velopharyngeal insufficiency associated speech difficulties) OR (Velopharyn* in*) OR (Velopharyn* dys*) OR (Palatal function) OR (Velopharyngeal Sphincter [Mesh]) OR		(Otitis Media with Effusion [Mesh]) OR (Otitis Media [Mesh]) OR (Otitis Media, Suppurative [Mesh]) OR (Hearing loss [Mesh]) OR (Hearing loss, Conductive [Mesh]) OR (Ear Diseases [Mesh]) OR (Otological disease) OR (Otologic* NEXT dis*) OR (Otiti* NEXT medi*) OR (Hearing loss)		(Cleft Palate surgery) OR (Cleft surgery) OR (Cleft palat* NEXT repair*) OR (Surgical cleft palate repair) OR (Surgical Flaps [Mesh]) OR (Posterior pharyngeal wall augmentation) OR (Buccinator myomucosal flaps) OR (Sphincter pharyngoplasty) OR (Furlow double-opposing Z-plasty) OR (Posterior pharyngeal flap) OR (Palatoplasty) OR (Palat*

	robotic) OR (Transoral robotic surgery)				(Communication disorder [Mesh]) OR (Nasal emission) OR (nasal turbulence) OR (hyponasality) OR (hypernasality) OR (Articulation disorder [Mesh]) OR (Nasal NEXT resonanc*) OR (Hypernasal* NEXT speech) OR (Hypernasal*) OR (Nasal NEAR air NEAR leak) OR (Nasal NEAR air NEAR escape) OR (Nasal NEXT turbulence) OR (Nasal rustle) OR (VPI) OR (Rhinolalia aperta)		OR (Conductive hearing loss)		NEAR surgery) OR (Cleft NEXT palat* NEAR surgery) OR (Cleft NEXT palat* NEAR repair) OR (Manual cleft palate surgery) OR (Manual NEAR cleft NEXT palat* NEAR surgery)
	**Number of results**		**Number of results**		**Number of results**		**Number of results**		**Number of results**
	9100		1051		4502		10642		3386
**Complete search string**	((Robotic assisted cleft palate surgery) OR (Robotic cleft palate surgery) OR (robotic cleft surgery) OR (robotic assisted cleft surgery) OR (Robotic Surgical Procedures [Mesh]) OR (Robotic) OR (Robotic assisted surgery) OR (Robotic surgery) OR (Robot) OR (Robot-assisted) OR (Transoral robotic) OR (Transoral robotic surgery)) AND ((Cleft Palate [Mesh]) OR (Cleft Soft Palate) OR (Cleft Hard Palate) OR (Cleft NEXT palat*)) AND ((Cleft Palate surgery) OR (Cleft surgery) OR (Cleft palat* NEXT repair*) OR (Surgical cleft palate repair) OR (Surgical Flaps [Mesh]) OR (Posterior pharyngeal wall augmentation) OR (Buccinator myomucosal flaps) OR (Sphincter pharyngoplasty) OR (Furlow double-opposing Z-plasty) OR (Posterior pharyngeal flap) OR (Palatoplasty) OR (Palat* NEAR surgery) OR (Cleft NEXT palat* NEAR surgery) OR (Cleft NEXT palat* NEAR repair) OR (Manual cleft palate surgery) OR (Manual NEAR cleft NEXT palat* NEAR surgery)) AND ((Velopharyngeal Insufficiency [Mesh]) OR (Speech Sound Disorder [Mesh]) OR (Speech [Mesh]) OR (Speech Intelligibility [Mesh]) OR (Speech Disorders) OR (Speech Acoustics) OR (Velopharyngeal insufficiency associated speech difficulties) OR (Velopharyn* in*) OR (Velopharyn* dys*) OR (Palatal function) OR (Velopharyngeal Sphincter [Mesh]) OR (Communication disorder [Mesh]) OR (Nasal emission) OR (nasal turbulence) OR (hyponasality) OR (hypernasality) OR (Articulation disorder [Mesh]) OR (Nasal NEXT resonanc*) OR (Hypernasal* NEXT speech) OR (Hypernasal*) OR (Nasal NEAR air NEAR leak) OR (Nasal NEAR air NEAR escape) OR (Nasal NEXT turbulence) OR (Nasal rustle) OR (VPI) OR (Rhinolalia aperta)) AND ((Otitis Media with Effusion [Mesh]) OR (Otitis Media [Mesh]) OR (Otitis Media, Suppurative [Mesh]) OR (Hearing loss [Mesh]) OR (Hearing loss, Conductive [Mesh]) OR (Ear Diseases [Mesh]) OR (Otological disease) OR (Otologic* NEXT dis*) OR (Otiti* NEXT medi*) OR (Hearing loss) OR (Conductive hearing loss))								
**Number of results**	0								
**Alternative search string**	((Robotic assisted cleft palate surgery) OR (Robotic cleft palate surgery) OR (robotic cleft surgery) OR (robotic assisted cleft surgery) OR (Robotic Surgical Procedures [Mesh]) OR (Robotic) OR (Robotic assisted surgery) OR (Robotic surgery) OR (Robot) OR (Robot-assisted) OR (Transoral robotic) OR (Transoral robotic surgery)) AND ((Cleft Palate [Mesh]) OR (Cleft Soft Palate) OR (Cleft Hard Palate) OR (Cleft NEXT palat*)) AND ((Cleft Palate surgery) OR (Cleft surgery) OR (Cleft palat* NEXT repair*) OR (Surgical cleft palate repair) OR (Surgical Flaps [Mesh]) OR (Posterior pharyngeal wall augmentation) OR (Buccinator myomucosal flaps) OR (Sphincter pharyngoplasty)*								
**Number of results**	1								
**Source/Database:**	Cochrane Library								

Search limits	
**Study type:**	
**Age range:**	
**Date range:**	
**Language:**	Dutch, English
**Other:**	

Supplemental Material S1: An overview of included and excluded publications

Included publications:

1. Smartt, J. M., Jr., Gerety, P., Serletti, J. M., & Taylor, J. A. (2013). Application of a Robotic Telemanipulator to Perform Posterior Pharyngeal Flap Surgery: A Feasibility Study. Plastic and Reconstructive Surgery, 131(4).
2. Khan, K., Dobbs, T., Swan, M. C., Weinstein, G. S., & Goodacre, T. E. (2016). Trans-oral robotic cleft surgery (TORCS) for palate and posterior pharyngeal wall reconstruction: A feasibility study. J Plast Reconstr Aesthet Surg, 69(1), 97-100.
3. Nadjmi, N. (2016). Transoral Robotic Cleft Palate Surgery. Cleft Palate Craniofac J, 53(3), 326-331.
4. Podolsky, D. J., Fisher, D. M., Wong Riff, K. W. Y., Looi, T., Drake, J. M., & Forrest, C. R. (2017). Infant Robotic Cleft Palate Surgery: A Feasibility Assessment Using a Realistic Cleft Palate Simulator. Plastic and Reconstructive Surgery, 139(2).
5. Téblick, S., Ruymaekers, M., Van de Casteele, E., Boudewyns, A., & Nadjmi, N. (2023). The effect of soft palate reconstruction with the da Vinci robot on middle ear function in children: an observational study. Int J Oral Maxillofac Surg, 52(9), 931-938.
6. Maguire, G., Tang, E., Looi, T., & Podolsky, D. (2025). Robotic Assisted Cleft Palate Repair Using Novel 3 mm Tools: A Reachability and Collision Analysis. IEEE Trans Biomed Eng, 72(7), 2085-2094.

Excluded publications:

1. Figueiredo, J., Melo, S., Carneiro, P., Moreira, A. M., Fernandes, M. S., Ribeiro, A. S., Guilford, P., Paredes, J., & Seruca, R. (2019). Clinical spectrum and pleiotropic nature of CDH1 germline mutations. J Med Genet, 56(4), 199-208.
2. Hammal, Z., Cohn, J. F., Wallace, E. R., Heike, C. L., Birgfeld, C. B., Oster, H., & Speltz, M. L. (2018). Facial Expressiveness in Infants With and Without Craniofacial Microsomia: Preliminary Findings. Cleft Palate Craniofac J, 55(5), 711-720.
3. Nadjmi, N. (2015). Transoral robotic cleft palate surgery (TORCS). International Journal of Oral and Maxillofacial Surgery, 44, e114-e115.
4. Tang, R., Zhang, C., Liu, B., Jiang, C., Wang, L., Zhang, X., Huang, Q., Liu, J., & Li, L. (2022). Towards an artificial peripheral nerve: Liquid metal-based fluidic cuff electrodes for long-term nerve stimulation and recording. Biosens Bioelectron, 216, 114600.
5. Yom, J., Palacios, J. F., Neuwirth, A., Atamian, E. K., Goldstein, T., & Bastidas, N. (2025). "Man vs. Machine: 3D Milling of Auricular Frameworks". Cleft Palate Craniofac J, 62(11), 1986-1991.
6. Gandhi, S. M., Patel, P., Carter, T., & Stutts, L. (2024). A 29-Year-Old Patient With Patau Syndrome: A Case Report on Medical Management. Cureus, 16(1), e51471.
7. Garcia, J. A., Najjar, W., Andari, D., Assaf, R. K., Annan, B., Johnson, A., Vyas, R., Swanson, J. W., & Hamdan, U. S. (2025). Ergonomics for Cleft Providers: A Systematic Review. Ann Plast Surg, 94(5), 612-622.
8. Li, Y., Cheng, J., Mei, H., Ma, H., Chen, Z., & Li, Y. (2019). CLPNet: Cleft Lip and Palate Surgery Support With Deep Learning. Annu Int Conf IEEE Eng Med Biol Soc, 2019, 3666-3672.
9. Mokhtar, J., Almarzooqi, S., Alhammadi, F., & Mendonca, D. A. (2025). Pilot Study: RoboticScope (Robotic Microscope)-assisted Primary Cleft Palate Surgery. Plast Reconstr Surg Glob Open, 13(5), e6744.

10. Varol, A., & Basa, S. (2009). The role of computer-aided 3D surgery and stereolithographic modelling for vector orientation in premaxillary and trans-sinusoidal maxillary distraction osteogenesis. Int J Med Robot, 5(2), 198-206.
11. Al Omran, Y., Abdall-Razak, A., Ghassemi, N., Alomran, S., Yang, D., & Ghanem, A. M. (2019). Robotics in Cleft Surgery: Origins, Current Status and Future Directions. Robot Surg, 6, 41-46.
12. Bonawitz, S. C., & Duvvuri, U. (2013). Robotic-assisted FAMM flap for soft palate reconstruction. The Laryngoscope, 123(4), 870-874.
13. Coelho, M. B., Peltz, T. S., Hunt, J. A., & Gianoutsos, M. (2024). Robotic surgery in plastic surgery: a review of its potential. Australasian Journal of Plastic Surgery, 7(1).
14. Kim, H., Cho, S.-u., & Kim, D. (2021). Robot-assisted surgeries in oral and maxillofacial area: a narrative review on the present, advantages and its future. Frontiers of Oral and Maxillofacial Medicine, 4.
15. Liu, H.-H., Li, L.-J., Shi, B., Xu, C.-W., & Luo, E. (2017). Robotic surgical systems in maxillofacial surgery: a review. International Journal of Oral Science, 9(2), 63-73.
16. Smartt, J. M., Jr., Gerety, P., & Taylor, J. A. (2014). Robotic Approaches to Palatoplasty and the Treatment of Velopharyngeal Dysfunction. Semin Plast Surg, 28(1), 32-34.
17. Whiteman, E., Rehman, U., Hussien, M., Sarwar, M. S., Harsten, R., & Brennan, P. A. (2025). Implementation of robotic systems in paediatric craniofacial and head and neck surgery: a narrative review of the literature. British Journal of Oral and Maxillofacial Surgery, 63(3), 165-173.
18. Dogan Ari, A. B., Turkyilmaz, A., Kolkiran, A., Tasdelen, E., & Kilic, E. (2025). Ectodermal dysplasias and isolated ectodermal anomalies: expanding the clinical and molecular spectrum in a cohort of 36 patients. European Journal of Pediatrics, 184(11), 667.
19. Irace, A. L., Shank, C., Adil, E. A., Cunningham, M. J., Kawai, K., Sideridis, G., & Rahbar, R. (2018). Changes in scope of procedures performed by pediatric otolaryngologists in the past decade. JAMA Otolaryngology - Head and Neck Surgery, 144(4), 322-329.
20. Mace, E., Solis, R., & Belcher, R. (2025). Surgery for Velopharyngeal Dysfunction. Facial Plastic Surgery Clinics of North America, 33(4), 581-593.
21. Maden Bedel, F., Balasar, O., Erol Aytekin, S., Keles, S., & Caksen, H. (2024). Lenz-Majewski syndrome and recurrent otitis media: Are they related or not? European Journal of Medical Genetics, 68, 104910.
22. Oommen, B. J., Raghunath, G., & Kuipers, B. (2006). Parameter learning from stochastic teachers and stochastic compulsive liars. IEEE transactions on systems, man, and cybernetics. Part B, Cybernetics : a publication of the IEEE Systems, Man, and Cybernetics Society, 36(4), 820-834.
23. Shen, S. C., & Li, H. Y. (2020). State-of-the Art in Reconstructive Palatal Surgery Techniques for Obstructive Sleep Apnea. Current Sleep Medicine Reports, 6(2), 67-75.
24. Zhang, A., Holdener, K., Stylianou, A., Spencer, J., & Lin, A. (2023). Development of a Hypernasality Screening Tool: An Innovative Approach using Mobile App, Crowdsourcing, and Artificial Intelligence. Cleft Palate Craniofacial Journal, 60(5 Supplement), 53.
25. Nguyen Dc Fau - Farber, S. J., Farber Sj Fau - Skolnick, G. B., Skolnick Gb Fau - Awad, M. M., Awad Mm Fau - Woo, A. S., & Woo, A. S. Abstract: Robotic-Assisted Cleft Palate Repair: A Feasibility Study. LID - 180-180. (2169-7574 (Electronic)).

Supplemental Material S2: Risk of bias assessment

1. Smartt, J.M., Jr., P. Gerety, and J.A. Taylor, Robotic Approaches to Palatoplasty and the Treatment of Velopharyngeal Dysfunction. Semin Plast Surg, 2014. 28(1): p. 32-4.

Because this study is a cadaveric feasibility study without patient outcomes or a comparator group, a formal RoB 2 assessment was not applicable. Methodological quality was evaluated according to IDEAL recommendations for early-stage surgical innovation studies.
This article is valuable as an early surgical innovation study, but its conclusions are highly susceptible to bias and cannot support claims that robotic palatal surgery improves patient outcomes.

Domain	Risk of Bias	Justification
Selection Bias	High	Only three cadaveric specimens were used. No description of specimen selection or representativeness.
Performance Bias	High	Procedures were performed by surgeons experienced with the technique and aware of study objectives. No control group or blinding.
Detection/Outcome Assessment Bias	High	Primary outcomes were subjective (improved exposure, minimal fatigue). No validated measurement instruments were used.
Attrition Bias	Low	No loss of specimens; all three procedures were completed successfully.
Reporting Bias	Moderate to High	The article emphasizes successful completion and perceived benefits while providing limited reporting of technical difficulties, failures, or adverse findings.
Confounding	High	No comparison with conventional surgery, making it impossible to determine whether outcomes were attributable to robotics or surgeon expertise.
External Validity (Applicability)	High risk of limited applicability	Cadaveric tissue does not replicate live surgical conditions such as bleeding, healing, airway management, tissue edema, speech outcomes, or complications.
Conflict of Interest Bias	Unclear	No conflicts of interest or industry funding are reported in the provided article excerpt.
Sample Size Bias	High	Extremely small sample (n=3) limits the reliability and precision of conclusions.

2. Khan, K., et al., *Trans-oral robotic cleft surgery (TORCS) for palate and posterior pharyngeal wall reconstruction: A feasibility study.* Journal of Plastic, Reconstructive & Aesthetic Surgery, 2016. 69(1): p. 97-100.

A formal RoB 2 or ROBINS-I assessment was not applicable because the study was a preclinical cadaveric/manikin feasibility study without patient participants. Methodological quality was therefore assessed using principles derived from the IDEAL framework for early-stage surgical innovation and general risk-of-bias domains (selection, detection, performance, reporting and external validity).
This study provides low-level evidence that trans-oral robotic cleft surgery is technically feasible in simulated and cadaveric settings. The evidence is highly susceptible to bias due to the absence of patients, controls, objective outcome measures, and comparative analyses. Therefore, the findings support technical feasibility only and do not provide evidence regarding clinical effectiveness, safety, or superiority over conventional cleft surgery.

Domain	Risk of Bias	Justification
Selection Bias	High	Only one manikin model and one adult cadaver were studied. No rationale for specimen selection or representativeness of cleft patients was provided.
Performance Bias	High	Investigators performed procedures while aware of the study hypothesis that robotics could improve access and visualization. No comparator procedures were performed using conventional techniques.
Detection Bias	High	Primary outcomes were subjective assessments of visualization and access, rated as "poor, fair, good or excellent." No objective validated measures were used.
Confounding	High	No control group exists. Improvements could reflect operator expertise, simulator characteristics, or study conditions rather than robotic technology itself.
Attrition Bias	Low	All planned simulations and cadaveric procedures were completed and reported.
Reporting Bias	Moderate–High	Positive findings are emphasized ("technically feasible", "short learning curve", "improved ergonomics"), while quantitative data are sparse and unsuccessful configurations are not fully described.
External Validity	High Risk of Limited Applicability	Results from a manikin and an adult cadaver cannot reliably predict outcomes in pediatric cleft patients undergoing live surgery. Tissue characteristics, bleeding, healing, airway issues, speech outcomes, and complications cannot be evaluated.
Conflict of Interest Bias	Low/Unclear	Authors report no conflicts of interest, and funding came from a research grant rather than industry sponsorship.
Sample Size Bias	High	Extremely limited sample (one manikin model and one cadaver) provides very weak evidence.

3. Podolsky, D.J., et al., *Infant Robotic Cleft Palate Surgery: A Feasibility Assessment Using a Realistic Cleft Palate Simulator.* Plast Reconstr Surg, 2017. 139(2): p. 455e-465e.

The IDEAL framework is most suitable because it is specifically designed to evaluate **early-stage surgical innovations in preclinical or simulator environments,** where the main concerns are feasibility, iteration, and safe progression—not clinical effectiveness or patient-level bias.

This study represents a well-conducted early-stage surgical innovation feasibility study (IDEAL Stage 1–2a) with moderate methodological quality. It provides credible evidence that robotic cleft palate repair is technically feasible within a realistic simulation environment. However, the absence of patient outcomes, objectively validated performance metrics, repeated testing, and clinical comparisons means that the findings should be interpreted as proof-of-concept evidence rather than evidence of clinical effectiveness, safety, or superiority over conventional cleft palate repair.

IDEAL Domain	Risk of Bias Judgment	Level of Risk	Justification
IDEAL Stage appropriateness (Stage 1–2a)	Appropriate stage classification	Low risk	Study correctly fits early IDEAL (Development/Early Exploration) as a preclinical feasibility evaluation
Innovation bias (novelty & enthusiasm effect)	Present	Moderate risk	High enthusiasm for robotic feasibility may influence interpretation toward positive framing of advantages
Selection bias (cases/tasks/operators)	Present	High risk	Expert operators likely used; no random selection of tasks or repeated independent operators reported
Performance bias (operator influence)	Present	High risk	Outcomes heavily dependent on the surgeon's skill and adaptation to the robotic system; no standardization of operator experience across trials
Confounding bias (device & instrumentation)	Severe	Critical risk	Major confounder: Si vs Xi comparison includes differences in instrument size, arm design, and ergonomics, not an isolated intervention effect
Measurement bias (outcome assessment)	Present	High risk	Outcomes (collisions, reachability, wrist orientation) are subjective, unblinded, and not based on validated scoring systems
Detection bias (assessment process)	Present	High risk	Outcome evaluation performed by the study team without blinding or independent adjudication
Reporting bias	Possible	Moderate–high risk	No protocol or pre-specified analysis plan; selective emphasis on favorable feasibility outcomes likely
Learning curve bias	Present	High risk	No formal accounting for learning effects or repeated practice sessions across systems
Simulation model bias (external validity limitation)	Present	High risk	Simulator lacks physiological realism (no bleeding, tissue variability, or patient safety constraints), limiting translational validity
Reproducibility bias	Moderate	Moderate risk	Procedural steps well described, but subjective scoring limits reproducibility across independent assessors
Ethical/clinical translation bias	Low	Low risk	Appropriate non-clinical stage avoids patient risk; ethical use of the simulation platform
IDEAL progression bias (overinterpretation risk)	Present	Moderate risk	Findings may be interpreted as supportive of clinical feasibility despite being preclinical simulator-only data

4. Téblick, S., et al., *The effect of soft palate reconstruction with the da Vinci robot on middle ear function in children: an observational study.* Int J Oral Maxillofac Surg, 2023. 52(9): p. 931-938.

ROBINS-I is suitable because this study is a non-randomized, retrospective comparative surgical intervention study with strong potential for confounding, selection bias, and time-related bias—precisely the type of evidence ROBINS-I was designed to critically appraise.
Although the study is methodologically strong for a retrospective surgical cohort (single surgeon, standardized protocol, longitudinal follow-up, and appropriate statistical modeling), the lack of randomization, temporal allocation bias, and potential confounding by indication substantially increase the risk of bias.

Domain	Risk of Bias Judgment	Rationale
1. Bias due to confounding	Serious	The study is non-randomized and treatment allocation (robot vs manual surgery) was based on shared decision-making after the introduction of the robotic system. Although baseline characteristics were statistically compared and appeared similar, important confounders (e.g., disease severity nuances, surgeon decision preferences, socio-environmental factors, prior ENT history beyond OME/VTE status, and unmeasured anatomical variation) were not fully controlled. Residual confounding is highly likely despite the inclusion of some covariates in statistical models.
2. Bias in selection of participants into the study	Moderate	Inclusion was retrospective and restricted to patients treated by a single surgeon within a defined period. However, the choice of robotic vs manual surgery was not randomized and coincided with the introduction of robotic surgery. This introduces potential selection bias linked to time period and parental preference (all parents reportedly preferred robotic surgery after its introduction), increasing the risk of systematic differences between groups.
3. Bias in classification of interventions	Low	Intervention status (robot-assisted vs manual dissection) is clearly defined and unlikely to be misclassified. Surgical records and procedural consistency by a single surgeon reduce classification errors.
4. Bias due to deviations from intended interventions	Low to Moderate	Surgical procedures were standardized (modified Furlow palatoplasty by a single surgeon), limiting deviation. However, lack of randomization and potential learning curve or temporal improvements in surgical technique (robot introduced in 2015) may have introduced systematic differences in care delivery over time.
5. Bias due to missing data	Moderate	Some missing outcome data were present (notably audiometry data at certain time points). Although statistical methods (mixed models, GEE) were used to mitigate missingness, the retrospective design increases the risk that missing data were not completely random. Loss to follow-up was minimal but not negligible.
6. Bias in measurement of outcomes	Low to Moderate	Outcomes (OME, ventilation tubes, audiometry) were measured using standard clinical protocols (otoscopy, tympanometry, pure tone audiometry). However, the lack of blinding of outcome assessors could introduce detection bias, especially for clinically subjective decisions such as diagnosis of OME or the decision to place ventilation tubes.
7. Bias in selection of the reported result	Moderate	Multiple statistical tests and time-point analyses were performed. While outcomes were clinically justified, the study does not explicitly state a pre-registered protocol, raising the possibility of selective reporting of favorable outcomes or time points.

5. Nadjmi, N., *Transoral Robotic Cleft Palate Surgery.* Cleft Palate Craniofac J, 2016. 53(3): p. 326-31.

This study primarily evaluates the feasibility, safety, and technical implementation of a novel robotic surgical technique in a small cohort of patients. As an early-stage surgical innovation study, it aligns with IDEAL Stage 2a (Development). Therefore, the IDEAL framework is considered more appropriate than ROBINS-I for methodological appraisal, as ROBINS-I is intended for evaluating comparative effectiveness and causal inference in non-randomized intervention studies rather than assessing the developmental stages of surgical innovation.
The study demonstrates successful preclinical preparation, transparent technical reporting, and acceptable early safety assessment. However, methodological limitations including a small sample size, lack of randomization, limited follow-up, and absence of learning-curve analysis, restrict the strength of the evidence. Consequently, the study provides preliminary feasibility evidence but does not establish comparative effectiveness or long-term clinical benefit.

IDEAL Domain	Assessment	Judgment	Comments
Stage Classification	New surgical technique evaluated in a small consecutive patient series following cadaver testing	Stage 2a (Development)	Appropriate stage designation; study focuses on feasibility and safety rather than effectiveness.
Innovation Description	Clear description of robotic setup, instrumentation, patient positioning, surgical steps, and cadaver testing	Low concern	Intervention is sufficiently described to permit replication.
Preclinical Development	Cadaver study conducted before clinical application	Low concern	Consistent with IDEAL recommendations for surgical innovation.
Patient Selection	10 consecutive patients included; selection criteria incompletely specified	Moderate concern	Consecutive recruitment reduces overt selection bias, but eligibility criteria are not fully detailed.
Transparency of Technique Evolution	Technique appears standardized; limited reporting of modifications during development	Moderate concern	IDEAL recommends explicit reporting of procedural refinements and learning effects.
Learning Curve Consideration	Surgeon experience and learning curve not formally evaluated	High concern	Particularly relevant for robotic surgery, where outcomes may improve with experience.
Comparator Group	30 matched controls treated manually	Moderate concern	Matching performed for age, sex, and diagnosis, but no randomization or adjustment for residual confounding.
Outcome Selection	Feasibility, operative time, complications, and hospital stay	Low concern	Outcomes are appropriate for an IDEAL Stage 2a study.
Outcome Measurement	Objective measures reported; follow-up ≥6 months	Low concern	Measurements are generally reliable and clinically relevant.
Safety Reporting	Intraoperative and postoperative complications reported	Low concern	Safety reporting is adequate for an early-stage innovation study.
Follow-up Completeness	Minimum 6 months follow-up (mean 8 ± 1 months)	Moderate concern	Adequate for feasibility but insufficient to assess speech outcomes, fistula formation, or long-term functional outcomes.
Generalisability	Single center, single surgical team	High concern	Findings may not be reproducible across surgeons or institutions.
Preparation for Next IDEAL Stage	Authors call for larger studies and further evaluation	Low concern	Appropriate progression toward IDEAL Stage 2b/3 studies.

6. <u>Maguire, G., et al., *Robotic Assisted Cleft Palate Repair Using Novel 3 mm Tools: A Reachability and Collision Analysis.* IEEE Trans Biomed Eng, 2025. 72(7): p. 2085-2094.</u>

This study carries a high risk of bias due to its reliance on a single expert operator and hand-built, rapidly wearing prototype hardware. It also presents a moderate risk of bias from unblinded, manual collision counting and confounding learning curve effects caused by fixed surgical sequencing.

**IDEAL Risk Domain**	**Risk Level**	**Assessment and Findings from Study**	**Implications for the Innovation**
1. Selection and Operator Bias	High	The physical simulator tasks and end-to-end repairs were performed by a single, highly experienced expert cleft surgeon.	The tool's usability metrics, collision reduction, and successful repair profiles may not generalize to trainees or surgeons with varying degrees of robotic experience.
2. Detection Bias *(Outcome Assessment)*	Moderate	Video recordings (ECM and external webcams) were reviewed manually to count tool-to-tool and tool-to-cavity collisions. Blinding was not explicitly stated, and the striking visual difference between 8 mm and 3 mm tools makes blinding highly difficult. However, image occlusion (visual field obstruction) was calculated semi-automatically via Python, which mitigates some bias.	There is a risk of subjective counting bias during video playback analysis due to the reviewer knowing which tool size was being evaluated.
3. Performance Bias *(Intervention Fidelity)*	High	The 3 mm prototypes were hand-built, subject to rapid cable wear, and required ongoing intraoperative maintenance, adjustments, or swapping during testing.	This mechanical inconsistency alters the tool's physical behavior mid-experiment, which likely skewed the collision variance recorded during the nasal and oral mucosal closures.
4. Confounding Bias *(Learning Curve Effect)*	Moderate	The end-to-end robotic repair on the simulator followed a fixed anatomical sequence (anterior to posterior for nasal closure; posterior to anterior for oral closure).	The surgeon’s increasing familiarity with the novel tool's custom kinematics over the course of the session is inherently confounded by the changes in depth of the anatomical target areas.
5. Reporting and Publication Bias	Low to Moderate	The authors transparently reported data that did not perfectly support their hypotheses (e.g., tool-tool collisions did not yield statistically significant correlation trends regarding oral depth; $p=0.277$ and $p=0.904$). They also cleanly documented the hardware failures and mechanical limitations (such as the 11.9° hysteresis).	High internal scientific integrity; data reporting is transparent regarding the prototype's current performance flaws.
